# Biochemical and hydrogen-deuterium exchange studies of the single nucleotide polymorphism Y649C in human platelet 12-lipoxygenase linked to a bleeding disorder

**DOI:** 10.1016/j.abb.2022.109472

**Published:** 2022-11-25

**Authors:** Michelle Tran, Rachel L. Signorelli, Adriana Yamaguchi, Eefie Chen, Michael Holinstat, Anthony T. Iavarone, Adam R. Offenbacher, Theodore Holman

**Affiliations:** a Department of Chemistry and Biochemistry, University of California Santa Cruz, Santa Cruz, CA, 95064, USA; b Department of Chemistry, East Carolina University, Greenville, NC, 27858, USA; c Department of Pharmacology, University of Michigan Medical School, Ann Arbor, MI, 48109, USA; d QB3/Chemistry Mass Spectrometry Facility, University of California Berkeley, Berkeley, CA, 94720, USA

## Abstract

Human platelet 12-lipoxygenase (h12-LOX) is responsible for the formation of oxylipin products that play an important role in platelet aggregation. Single nucleotide polymorphisms (SNPs) of h12-LOX have been implicated in several diseases. In this study, we investigate the structural, dynamical, and functional impact of a h12-LOX SNP that generates a tyrosine-to-cysteine mutation at a buried site (Y649C h12-LOX) and was previously ascribed with reduced levels of 12(S)-hydroxyeicosa-tetraenoic acid (12S-HETE) production in isolated platelets. Herein, *in vitro* Michaelis-Menten kinetics show reduced catalytic rates for Y649C compared to WT h12-LOX at physiological or lower temperatures. Both proteins exhibited similar melting temperatures, metal content, and oligomerization state. Liposome binding for both proteins was also dependent upon the presence of calcium, temperature, and liposome composition; however, the Y649C variant was found to have lowered binding capacity to liposomes compared to WT at physiological temperatures. Further, hydrogen-deuterium exchange mass spectrometry (HDX-MS) experiments revealed a regional defined enhancement in the peptide mobility caused by the mutation. This increased instability for the mutation stemmed from a change in an interaction with an arched helix that lines the substrate binding site, located ≥15 Å from the mutation site. Finally, differential scanning calorimetry demonstrated a reduced protein (un)folding enthalpy, consistent with the HDX results. Taken together, these results demonstrate remarkable similarity between the mutant and WT h12-LOX, and yet, subtle changes in activity, membrane affinity and protein stability may be responsible for the significant physiological changes that the Y649C SNP manifests in platelet biology.

## 1. Introduction

Platelet activation is essential to produce a platelet plug to restore hemostasis after injuries, however, uncontrolled platelet activation can also lead to the formation of an occlusive thrombus resulting in myocardial infarction or stroke. The importance platelets play in thrombotic disorders has been established through the decrease of thrombotic events with the use of antiplatelet therapeutics [1,2]. In addition, the role of platelets in aggregation is underscored by single nucleotide polymorphisms (SNPs) which can affect platelet biology significantly [3-7].

Human platelet 12-lipoxygenase (h12-LOX) is a non-heme, iron-containing enzyme that catalyzes the oxidation of polyunsaturated fatty acids (PUFAs) with *cis*-cis-1,4-pentadiene moieties, such as arachidonic acid (AA). These PUFA substrates are found in the phospholipid bilayer and can be released from the membrane by cleavage with a phospholipase [8]. h12-LOX is proposed to associate with the bilayer upon calcium release and react with the free PUFAs that are associated with the bilayer. It has been well documented that calcium leads to the translocation of h12-LOX from the cytosol to the membrane where free AA is associated with the bilayer [9-11]; however, h12-LOX has poor reactivity with PUFA molecules covalently attached to the phospholipid bilayer [12].

The exact mechanism by which h12-LOX activates platelets is unknown. It has been postulated that 12(S)-hydroxyeicosatetraenoic acid (12S-HETE) activates NADPH oxidase to form reactive oxygen species, molecules known to lead to platelet activation, but it is not known whether NADPH oxidase is required for h12-LOX activation [13]. 12S-HETE has also been hypothesized to bind to GPR31, an orphan GPCR, causing a prothrombotic result [14]. Studies inhibiting the formation of 12S-HETE showed a decrease in thrombus growth, vessel occlusion, and plug formation in mice further supporting h12-LOX’s prothrombotic behavior. It has also been strongly suggested that 12-HETE leads to tissue-factor-dependent thrombin generation due to its esterification into the membrane [15-17]. Further, transgenic mice deficient in h12-LOX were not able to undergo normal platelet aggregation [18]. With regards to possible physical protein-protein interactions, strong evidence has shown that h12-LOX activates phospholipase C2, phosphokinase C, induces αIIbβ3 aggregation, and increases calcium concentrations [18].

**Fig. 1.**
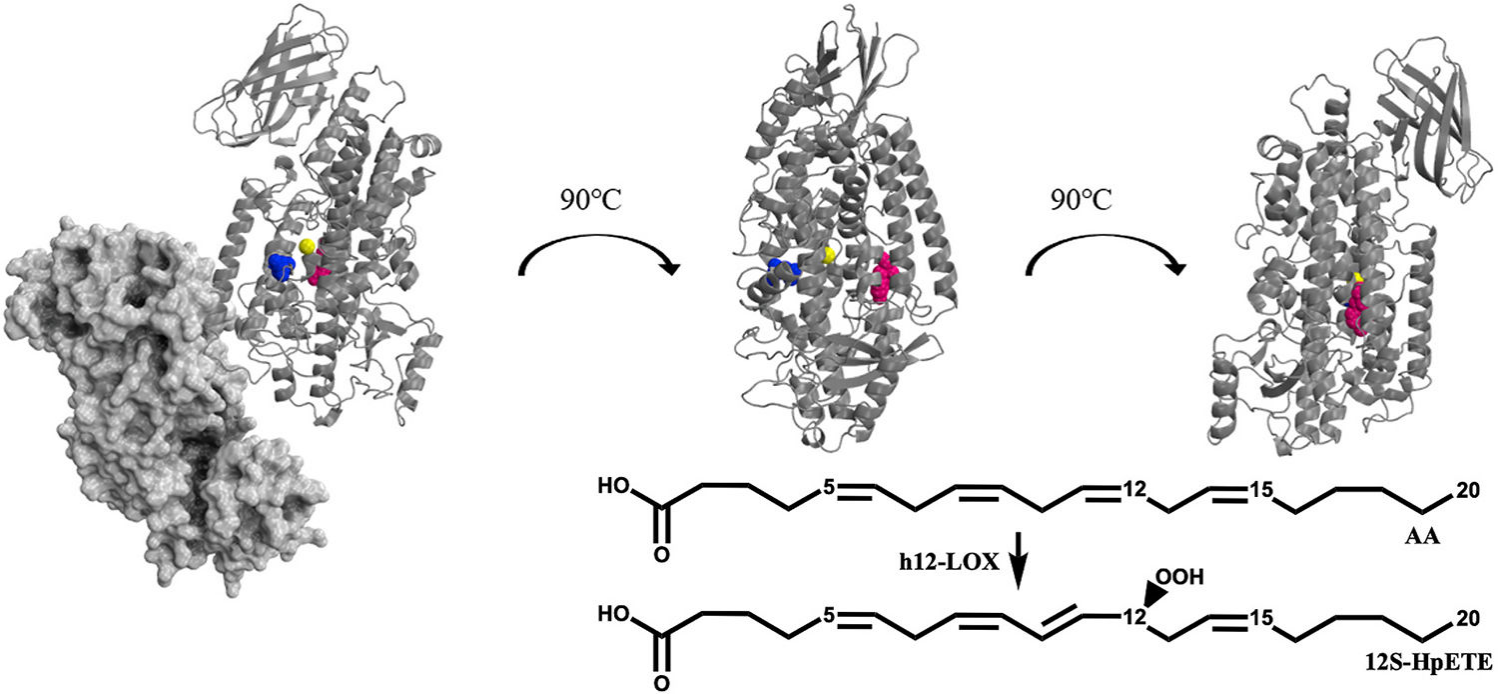
The enzymatic reaction of h12-LOX with AA, producing 12S-HpETE is shown (Insert) and a h12-LOX structural model [19] with Y649 highlighted in pink, L407 (a critical active site residue) highlighted in blue and the active site iron in yellow. The proposed solution-state dimer is shown on the left, with one monomer shown as a dimmed grey space-filling model and the other monomer shown as a grey ribbon model. The other two images are of only the cartoon monomer, for clarity of presentation.

SNPs in the wild-type (WT) h12-LOX coding region have been correlated with diseases such as human congenital toxoplasmosis [20], breast cancer [21], schizophrenia [22], osteoporosis [23], and early onset menopause [24]. However, a majority of the discovered SNPs, such as N322S, E259K, Q261R, and D134H, have been found on the surface of h12-LOX and their ability to produce 12-HpETE is comparable to WT h12-LOX (101 ± 23%, 60 ± 18%, 91 ± 10%, and 109 ± 36%, respectively) [25]. Recently, a study documenting a family with a dominantly inherited bleeding diathesis was linked to a h12-LOX SNP, Y649C [26]. A patient with this mutation experienced heavy menstrual bleeds and needed platelet transfusion during childbirth. In addition, both daughters of the patient developed purpura on their lower limbs. Unlike most documented SNPs of h12-LOX, Y649C is buried within the catalytic domain (Fig. 1) and Y649C demonstrated a 25–35% loss of 12S-HETE production in patient-isolated platelets [26]. However, it was unclear why the mutation afforded this change in 12S-HETE production [26]. Due to the critical role 12S-HETE plays in regulating platelet aggregation and thrombosis, studying the correlation between the structure and function of the mutation is pertinent to understanding its role in the platelet and may potentially enable us to provide a more accurate and precise treatment to patients. In the current study, we examine the role of this mutation on the enzyme’s temperature-dependent kinetic properties and membrane binding capacity. The regional peptide flexibility of the SNP was also investigated with the goal of relating it to the protein folding enthalpy and catalytic activity. In combination, our cumulative results shed important new light on the structural, functional, and dynamical impact of this SNP of h12-LOX and its potential relationship to the pathological condition.

## 2. Experimental procedures

### 2.1. Chemicals

Fatty acids used in this study were purchased from Nu Chek Prep, Inc. (MN, USA). Deuterium oxide (99.9%) was purchased from Cambridge Isotope Laboratories (Tewksbury, MA, USA). All other solvents and chemicals were reagent grade or better and were used as purchased without further purification.

### 2.2. Site-directed mutagenesis, protein expression/purification, and metal content

The h12-LOX mutation, Y649C, was introduced using the same residue numbering convention as used in UniProt for the h12-LOX sequence (accession number P18054). The online QuikChange Primer Design tool (http://www.genomics.agilent.com/primerDesignProgram.jsp) from Agilent Technologies (CA, USA) was used to design the primers for the Y649C mutant. The mutation was introduced using a QuikChange^®^ II XL site-directed mutagenesis kit from Agilent Technologies by following the instructions in the provided protocol. The mutation was confirmed by sequencing the LOX insert in the pFastBac3.2 shuttle vector (Eurofins Genomics, KY, USA).

Expression of the N_t_-6His-tag recombinant enzymes was performed in an Sf9 system, with chromatographic purification and enzymatic activity assays performed as described previously [27]. All proteins were aliquoted and stored in 25 mM HEPES (pH 8) and samples from the same purification batch were used for all analyses. The reaction time course of enzymatic activity was monitored continuously at 234 nm using a PerkinElmer Lambda 45 UV/Vis spectrophotometer to observe the production of conjugated dienes (epsilon at 234 nm = 27,000 mM^−1^ cm^−1^ for 12S-HpETE) in a cuvette containing a 2 mL solution of 25 mM HEPES (pH 8), 10 μM AA and 20–30 μg of WT or Y649C h12-LOX. Iron content was determined by inductively coupled plasma mass spectrometry (ICP-MS), using an internal Co standard and external standardized Fe solutions. Iron concentrations were compared with standardized iron solutions and all kinetic data were normalized to the iron content. The Bradford assay, with bovine serum albumin (BSA) as the protein standard, was used to determine the protein concentration.

### 2.3. Steady-state kinetics and temperature stability

h12-LOX reactions were performed at 22, 37, and 42 °C in a 1 cm^2^ quartz cuvette containing 2 mL of 25 mM HEPES (pH 7.5) with substrate (docosahexaenoic acid (DHA) and AA). DHA and AA concentrations were varied from 0.25 to 10 μM. Concentrations of DHA and AA were determined by measuring the amount of oxylipins produced from complete reaction with soybean lipoxygenase-1 (SLO-1). Concentrations of oxylipins were determined by measuring the absorbance at 234 nm. Reactions were initiated by the addition ~20 μg of WT and ~30 μg of Y649C h12-LOX and were monitored on a PerkinElmer Lambda 45 UV/Vis spectrophotometer. Product formation was determined by the increase in absorbance at 234 nm for oxylipin products (*ε*_234nm_ = 25,000 M^−1^ cm^−1^), within the first 20 s of the reaction where no enzyme inactivation was observed. KaleidaGraph (Synergy) was used to fit initial rates (at less than 20% turnover), to the Michaelis-Menten equation for the calculation of kinetic parameters. For the stability assay, all reactions were conducted using a Cary-UV Vis spectrophotometer, which was set to 22 and 37 °C, with 25 mM HEPES buffer (pH 8) and stirred continuously. Stocks of WT and Y649C at 133 nM and 200 nM respectively were incubated at 22 and 37 °C, with 6000 μL being withdrawn at each time point. The fastest rates over 15 s intervals were plotted versus the enzyme incubation time at 37 °C.

### 2.4. Enzymatic products determination and competitive substrate specificity by LC-MS

To determine the products formed, WT and the Y649C enzymes were reacted with 10 μM of AA in 25 mM HEPES buffer (pH 7.5) for 10 min, in triplicate. The reactions were quenched with 1% glacial acetic acid and extracted three times with dichloromethane (DCM). The products were then reduced with trimethylphosphite and evaporated under a stream of nitrogen gas. The reaction products were reconstituted in methanol and analyzed via liquid chromatography-tandem mass spectrometry (LC-MS/MS). Chromatographic separation was performed using a Sciex Excion LC system, using a C18 column (Phenomenex Kinetex, 4 μm, 150 × 2.0 mm). Mobile phase A consisted of water with 0.1% (v/v) formic acid, and mobile phase B was acetonitrile with 0.1% formic acid. The flow rate was 0.4 mL/min with initial conditions (15% B) maintained for 0.75 min. Mobile phase B was held at 15% over 1 min and then ramped to 30% over 0.75 min, to 47% over 2 min, to 54% over 1.5 min, to 60% over 4.5 min, to 70% over 4.5 min, to 80% over 1 min, to 100% over 1 min, held at 100% for 2 min, and finally returned to 15% to equilibrate for 2 min. The chromatography system was coupled to a Sciex PDA and x500B qTOF MS. Analytes were ionized through electrospray ionization with a −4.0 kV spray voltage and 50, 50, and 20 PSI for ion source gases 1 and 2 and the curtain gas, respectively. The CAD gas was set to 7, while the probe temperature was 550 °C. DP was −60 V, and CE was set to −10 V with a 5 V spread. MS2 acquisition was performed using SWATH, and mass lists containing the following *m/z* ratios of 343.2 ± 0.5 (HDHAs) and 359.2 ± 0.5 (HETEs) were used. All analyses were performed in negative ionization mode at the normal resolution setting. Matching retention times, UV spectra, and fragmentation patterns to known standards with at least six common fragments were used to identify the products. For the competitive substrate specificity assay, a similar procedure to product formation was used. A mixture of 10 μM AA and 10 μM DHA was incubated with enzyme, with the reaction being quenched with 1% glacial acetic acid after a third of the total substrate turnover.

### 2.5. IC_50_ determination

IC_50_ values for the h12-LOX specific inhibitor, ML355, against WT and Y649C h12-LOX were determined in the same manner as the steady-state kinetic values. The reactions were carried out in 25 mM HEPES buffer (pH 8.00), 0.01% Triton X-100, and 10 μM AA. IC_50_ values were obtained by determining the enzymatic rate at six inhibitor concentrations and plotting rate against their inhibitor concentration, followed by a hyperbolic saturation curve fit. The data used for the saturation curve fits were performed in duplicate or triplicate, depending on the quality of the data. Triton X-100 was used to ensure proper solubilization of the inhibitor.

### 2.6. Oligomeric state determination

Size exclusion chromatography (SEC) was performed on the affinity-purified proteins using an AKTA Pure system. After centrifugation at 13,000 rpm for 5 min (to sediment any Debris), the purified protein was loaded onto a SuperdexTM 75, 10/300 GL column (GE Healthcare), preequilibrated with 25 mM HEPES (pH 8) at a flow rate of 0.3 mL/min. The time of elution of WT and Y649C was compared with a gel filtration standard containing bovine thyroglobulin (MW 670 kDa), bovine γ-globulin (158 kDa), chicken ovalbumin (44 kDa), ribonuclease A type I-A from bovine pancreas (17 kDa) and *p*-aminobenzoic acid (1.35 kDa).

### 2.7. Protein melting temperature determination with circular dichroism

Thermal denaturation spectra were measured on a CD spectrophotometer (J1500, JASCO, Inc., Easton, Maryland) using a 1 mm quartz cuvette in the 180–300 nm spectral region and the 25–81 °C temperature range. The temperature was controlled using a Peltier thermostatted cell holder, the thermal ramp rate was 0.6 °C/min, and the step size was 2 °C. Spectra were measured with a scan speed of 100 nm/min every 0.1 nm using a 4 nm bandwidth and a data integration time of 4 s. A 25 mM sodium phosphate buffer (pH 7.5) background CD spectrum was subtracted from each 10 μM WT or Y649 h12-LOX temperature-dependent CD spectrum. Phosphate buffers lacking sodium chloride were used for CD because chloride ions and common organic buffers, such as HEPES, interferes with CD signal in the UV region. Two to three sets of thermal melts were acquired for each of the WT and Y649 h12-LOX.

### 2.8. Synthetic liposome preparation and H12-LOX binding

Lipid suspensions were prepared from commercial sources with the following molar ratios; 60:30:9.9:0.1 DOPC:DOPE:DOPA:DSPE-PEG (DOPE); 60:30:9.9:0.1 DOPC:DOPS:DOPA:DSPE-PEG (DOPS); 99.9:0.1 DOPC: DSPE-PEG (DOPC). Each lipid mixture was dissolved in chloroform and the solutions were left under N_2_ for 20 min and placed in a vacuum chamber for at least 12 h at room temperature to remove all traces of solvent. Lipid mixtures were dissolved in 25 mM HEPES buffer (pH 8) to a concentration of 10 mg/mL and incubated in glass vials on a tube rotator for 1 h to facilitate homogenization. Liposomes were created using the literature protocol [28], using a 100 nm filter. For liposome membrane binding assays conducted at 37 °C, liposomes were extruded at 37 °C and remained at that temperature for the assay. Liposome suspension volumes were adjusted to have a final concentration of 10 mg/mL and their size were determined by dynamic light scattering (DLS).

Liposome suspensions were placed on a DynaMag^™^-2 for 15 min at room temperature to bind the liposomes. Supernatant was removed and 1 mL of 25 mM HEPES buffer (pH 8) was added. This washing step was repeated five times. When calcium (10 μM) was added, it was stirred for 15 min before use. WT (30 μg) or Y649C (30 μg) were added to 1 mL of a 10 mg/mL liposome suspension. The sample was rocked over ice for 10 min and then placed on a DynaMag^™^-2 for 10 min 25 μL of the supernatant was saved, and the beads were resuspended to a final volume of 1 mL. 25 μL of the resuspended beads were saved and the saved samples were subjected to SDS-PAGE. Using ImageLab, ratios of the supernatant to pellet were determined. All conditions were done in triplicate. In parallel, the remainder of the supernatant was added to a cuvette, its volume raised to 2 mL, and the sample tested for enzymatic activity with 10 μM AA. Rates of reactions were determined using the same protocol as used for steady-state kinetics.

### 2.9. HDX-MS of h12-LOX

Aliquots of WT or Y649C h12-LOX (3–5 mg/mL) were thawed and were diluted 10-fold (5 μL into 45 μL) in 10 mM HEPES, 150 mM NaCl, 5 mM dithiothreitol (DTT), pD 7.4 D_2_O (99% D) buffer (corrected; pD = pH_read_ + 0.4). Samples were incubated randomly at 10 time points (0, 10, 20, 45, 60, 180, 600, 1800, 3600, and 7200 s) at 10 and 25 °C using a water bath. For each variant and temperature, time points were randomized and prepared once (see Table S1). At the designated incubation time, all samples were then treated identically; the samples were rapidly cooled (5–6 s in a −20 °C bath) and acid quenched (to pH 2.4, confirmed with pH electrode, with 0.32 M citric acid stock solution to 90 mM final concentration). Procedures from this point were conducted near 4 °C. Prior to pepsin digestion, guanidine HCl (in citric acid, pH 2.4) was mixed with the samples to a final concentration of 0.5 M h12-LOX HDX samples were digested with pre-equilibrated (10 mM citrate buffer, pH 2.4), immobilized pepsin for 2.5 min. The peptide fragments were filtered with spin cups (cellulose acetate) by centrifugation for 10 s at 4 ° C, to remove pepsin. Samples were flash frozen immediately in liquid nitrogen and stored at −80 °C until data collection.

Deuterated, pepsin-digested samples of h12-LOX were analyzed using a 1200 series LC system (Agilent, Santa Clara, CA) that was connected in-line with the LTQ Orbitrap XL mass spectrometer (Thermo), as described by our laboratory previously [19,29]. Mass spectral data corresponding to the HDX measurements were analyzed using HDX Workbench [30]. The percent deuterium incorporation was calculated for each of these peptides, taking into account the number of amide linkages (excluding proline residues), and the calculated number of deuterons incorporated. The values were normalized for 100% D2O and corrected for peptide-specific back-exchange, HDX% = (observed, normalized extent of deuterium incorporation {in percent})/(1-{BE/100}) [31]. Back-exchange values ranged from 2 to 53%, for an average value of 22% (Supporting Information, HDX Summary Table; Table S1). The resulting data were plotted as deuterium exchange versus time using Igor Pro software.

### 2.10. Protein stability determined by differential scanning calorimetry (DSC)

DSC experiments were performed on the WT and Y649C h12-LOX variants using a NanoDSC microcalorimeter from TA Instruments. Protein samples were dissolved at 20–30 μM concentrations in 50 mM HEPES, 150 mM NaCl, pH 7.5. For a given experiment, the temperature was scanned from 30 to 90 °C, with a 1 °C/min ramp rate at constant pressure (3 atm). Experiments were performed in heating only mode because h12-LOX denatures and does not reversibly refold. The raw data was analyzed using NanoAnalyze software from TA instruments. Thermodynamic data was determined from Gaussian fit models of the raw data that had been corrected by using a fourth-order polynomial baseline. Each experiment was run in duplicate.

## 3. Results and discussion

### 3.1. 1–3.2 protein purification and expression

Using standard protein purification methods as used previously [27], the purity of WT and Y649C h12-LOX were assessed by SDS-PAGE gels to be greater than 90%. The metal content was determined to be 22% for WT and 20% for Y649C by inductively coupled plasma mass spectrometry (ICP-MS). All kinetic data were normalized to the iron content, and the protein concentrations were determined by Bradford assay with a bovine serum albumin protein standard.

#### 3.1.1. Determining k_cat_ and k_cat_/K_M_ for WT and Y649C H12-LOX at 22 °C, 37 °C, and 42 °C

**Fig. 2.**
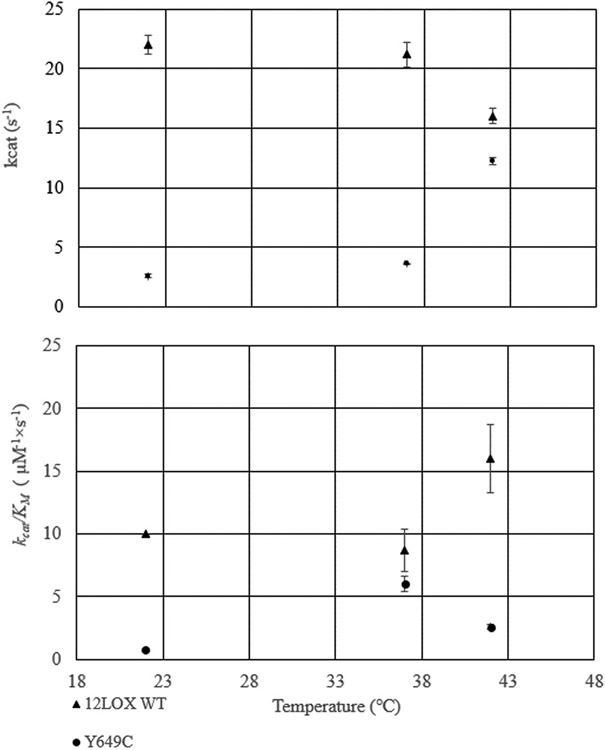
*k*_*cat*_ and *k*_*cat*_/*K*_*M*_ values for WT (triangles) and Y649C (circles) at 22 °C 37 °C. and 42 °C.

It was proposed that the activity of Y649C h12-LOX was markedly lower than WT h12-LOX, which manifested in the disease presentation [10]. Based on this hypothesis, the *in vitro* kinetic properties of the two enzymes were determined and compared (Table 1, Fig. 2). The *k*_*cat*_ for WT and Y649C with AA at 22 °C was 22 ± 4 and 2.6 ± 6 s^−1^, respectively, with the WT values correlating well with our previous work [32-37]. The *k*_*cat*_/*K*_*M*_ for WT and Y649C was 10 ± 0.2 and 0.76 ± 0.4 μM^−1^ s^−1^, respectively. These data demonstrate that the *k*_*cat*_ and *k*_*cat*_/*K*_*M*_ with AA for WT h12-LOX is ~10-fold greater than that of Y649C at 22 °C; however, the functional temperature in the human body is 37 °C. Therefore, the temperature dependences of *k*_*cat*_ and *k*_*cat*_/*K*_*M*_ were determined with AA at 37 °C and 42 °C. At 37 °C, the *k*_*cat*_ for WT and Y649C was 21 ± 1 and 3.6 ± 0.1 s^−1^, respectively. The *k*_*cat*_/*K*_*M*_ for WT and Y649C was 8.7 ± 2 and 6 ± 0.6 μM^−1^ s^−1^, respectively.

At 42 °C, the *k*_*cat*_ for WT h12-LOX and Y649C with AA was 16 ± 0.6 and 12 ± 0.3 s^−1^, respectively. The *k*_*cat*_/*K*_*M*_ for WT and Y649C was 16 ± 3 and 2.6 ± 0.2 μM^−1^ s^−1^, respectively. The kinetics for WT displayed relatively constant kinetic values from 22 °C to 37 °C, but a decrease in *k*_*cat*_ and an increase in *k*_*cat*_/*K*_*M*_ at 42 °C. The *k*_*cat*_ of Y649C also was relatively constant from 22 °C to 37°, but increased at 42 °C. The *k*_*cat*_/*K*_*M*_ of Y649C increased at 37 °C and then subsequently decreased at 42 °C. Overall, the kinetic parameters of WT are significantly greater than those of Y649C, with the *k*_*cat*_ of WT being over six-fold greater than that of Y649C at 37 °C, though the *k*_*cat*_/*K*_*M*_ values are comparable at physiological temperature. It is unlikely that these results are due to enzyme instability because the *k*_*cat*_/*K*_*M*_ value for WT increases, while that of Y649C decreases at 42 °C, which is opposite of the temperature dependence of their enzyme activity (*vide infra*).

The functional stability of the two enzymes at 22 and 37 °C was investigated by assessing the initial rates as a function of time at both 22 and 37 °C incubations. At 22 °C, both WT and Y649C retained over 90% activity after 120 min. Surprisingly, WT lost activity significantly faster than Y649C at 37 °C. WT lost 75% of its activity after only 200 s, whereas it took 3500 s for the activity of Y649C to drop 75% (Supporting Information, Fig. S1).

#### 3.1.2. Determining *k*_*cat*_ and *k*_*cat*_/*K*_*M*_ for WT h12-LOX and Y649C with DHA 22 °C

DHA oxylipins are predominately anti-inflammatory mediators, and AA oxylipins are mainly pro-inflammatory mediators [38], therefore the kinetics for the reaction of Y649C h12-LOX with DHA were investigated at 22 °C. The *k*_*cat*_ for the reactions of WT h12-LOX and Y649C with DHA was 11 s^−1^ and 1.2 s^−1^ respectively, while the *k*_*cat*_/*K*_*M*_ for WT and Y649C was 7.7 s^−1^ μM^−1^ and 0.96 s^−1^ μM^−1^, respectively. These data demonstrate that the WT variant is a more effective catalyst than Y649C with DHA, consistent with the kinetic trends with AA as the substrate, and that both WT and Y649C react with AA preferentially over DHA. To confirm this result, competitive kinetics were also performed (Table 2). WT demonstrated a preference for AA over DHA, producing 65% AA-oxylipins and 35% DHA-oxylipins, when both AA and DHA were present in the same reaction vessel. Y649C had a similar profile producing 63% AA-oxylipins and 37% DHA-oxylipins. There was approximately double the amount of oxylipins formed from AA than DHA which is consistent with the two-fold increase in *k*_*cat*_ and *k*_*cat*_/*K*_*M*_ between AA and DHA for both WT and Y649C [34,35,37].

### 3.2. Product profile and substrate specificity of AA and DHA with WT h12-LOX and Y649C

Given the significant role that the oxylipin products of h12-LOX play in platelet aggregation, the product profile for the reactions of Y649C h12-LOX and WT with both AA and DHA were investigated. With AA as substrate, WT and Y649C produced mostly the 12-oxylipin with 12- HETE:15-HETE ratios of 88:12 and 81:19, respectively. For DHA, the 14- oxylipin was the major product for both WT and Y649C, with 14-HDHA: 17-HDHA:20-HDHA: 11-HDHA ratios of 76:11:0:13 and 74:8:8:10, respectively (Table 3), which are consistent with previous work [34,35,37]. Thus, there was no significant difference between the product profile for these h12-LOX variants with either substrate.

### 3.3. IC_50_ determination with WT h12-LOX and Y649C

ML355 is a potent/selective h12-LOX inhibitor [39] which binds to its active site and thus its IC_50_ would be sensitive to structural changes in the active site. The IC_50_ values of ML355 were determined with both WT and Y649C and found to be 0.9 ± 0.2 μM (91 ± 5% maximum inhibition) and 1.1 ± 0.2 μM (90 ± 4% maximum inhibition), respectively. Since there was no significant difference between the IC_50_ values of WT and Y649C, the data indicates minimal change in the active site for inhibitor recognition (Table 4). The inhibitor data of WT are consistent with previous reports [39,40].

### 3.4. Oligomeric states of WT h12-LOX and Y649C

It has previously been shown that WT h12-LOX remains mainly in the dimeric state [19]. The oligomeric state of WT and Y649C were determined by size exclusion chromatograph (SEC). No significant difference was observed in the elution volume. Therefore, both remained in the dimer form, supporting that the Y649C mutation does not change the oligomeric state of h12-LOX (Supporting Information, Fig. S2).

### 3.5. Protein melting point determination of WT h12-LOX and Y649C by circular dichroism

To investigate the effects this mutation might have on the structural temperature dependence of h12-LOX, circular dichroism (CD) spectroscopy was used to determine the melting temperature of both WT and Y649C. The temperature-dependent protein unfolding traces were obtained by averaging the measured CD spectral data (Supporting Information, Fig. S3) from 205 to 225 nm. This spectral range encompasses the wavelengths absorbed by both major secondary structures (helix and sheet). By fitting the thermal traces to three-parameter sigmoidal functions, the temperature midpoints (T_m_) were determined to be 48 ± 0.5 °C for WT and 48 ± 1 °C for Y649C. These data dismiss any significant change in the melting of the secondary structure as the origin of the kinetic properties emerging from the replacement of the tyrosine residue at position 649 with cysteine.

### 3.6. Synthetic membrane association of WT H12-LOX and Y649C at 22 °C and 37 °C

**Fig. 3.**
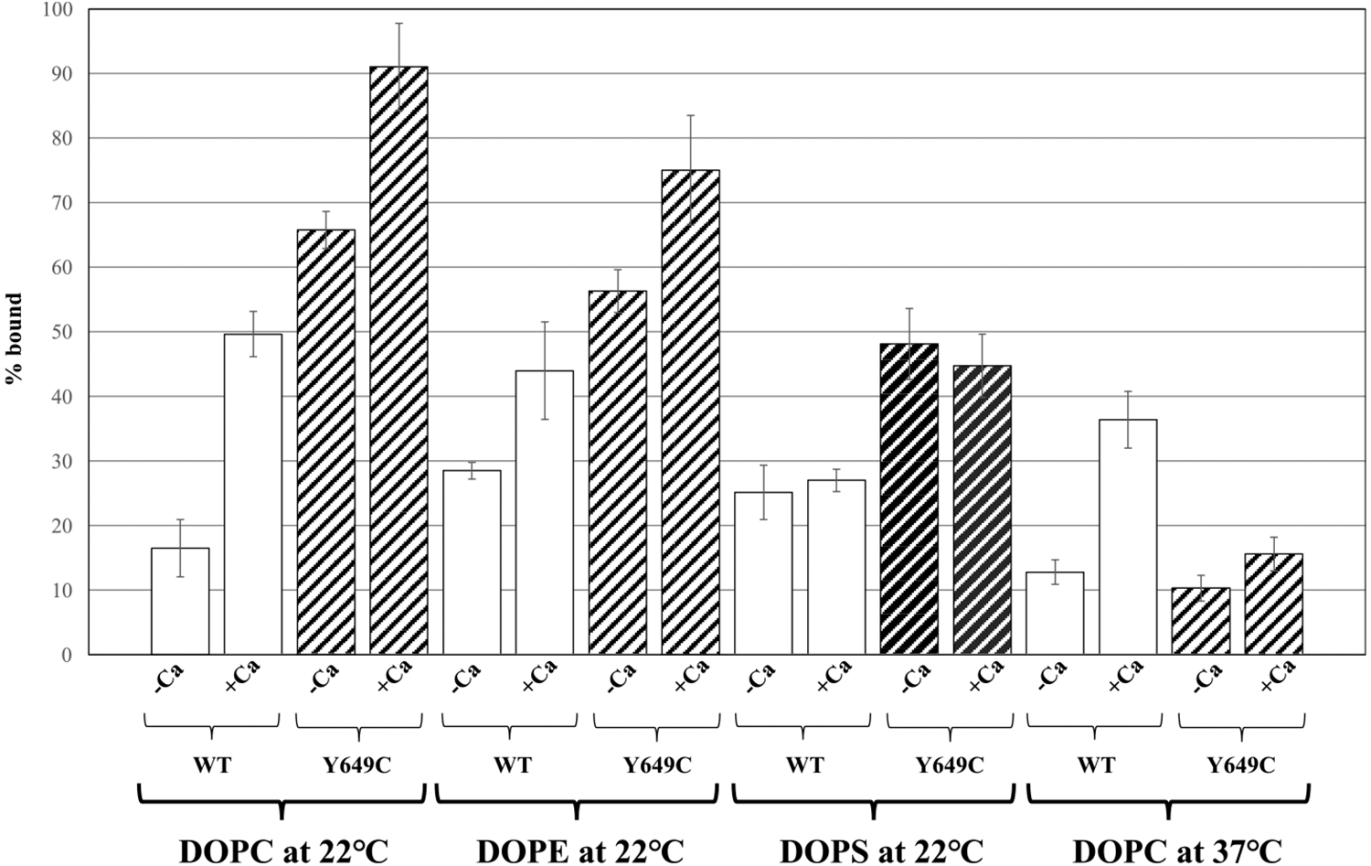
Percent of WT h12-LOX or Y649C bound to DOPC, DOPE, and DOPS liposomes at 22 °C and DOPC at 37 °C. Membrane binding assays were conducted in 25 mM HEPES (pH 7.5) at room temperature in either the absence or presence of 10 μM calcium chloride.

In the presence of DOPC liposomes (at 22 °C), 16 ± 4% of WT h12-LOX was bound to the membrane in the absence of calcium, and 50 ± 3% was bound in the presence of calcium. For Y649C, 66 ± 3% was bound in the absence of calcium; this value increased to 91 ± 7% when calcium was added. A similar trend was observed with DOPE liposomes for both proteins (Fig. 3), though the impact of calcium addition did not have as significant an effect on h12-LOX association with DOPE liposomes compared to DOPC. With DOPS liposomes, more Y649C associated with the liposomes than WT, consistent with DOPC and DOPE; however, there was no effect from addition of 10 μM CaCl_2_ for either protein (Fig 3). These data demonstrate that in general Y649C has a higher binding capacity to synthetic liposomes than WT at 22 °C and that calcium increases that affinity for both WT and Y649C, with DOPC and DOPE having the largest effect. This has been previously shown for WT h12-LOX with liposomes of comparable composition [10,11].

The temperature effect for DOPC liposome binding was investigated at 37 °C. At this temperature, 13 ± 2% of WT associated with the liposome, which increased to 36 ± 4% when calcium was added. For Y649C, 10 ± 2% was bound, which only increased to 16 ± 3% when calcium was added (Fig. 3). These data indicate that the binding affinity of WT is not significantly affected by the increase of temperature, which agrees with past literature [11]. However, the binding affinity of Y649C is significantly reduced at 37 °C and is nearly insensitive to the presence of calcium ions.

### 3.7. HDX-MS of Y649C h12-LOX

Based on an *in silico* model [19], the site of mutation (Y649C) is 15–18 Å from the catalytic iron center and nearly 30 Å (Cα-Cα) from a conserved active site leucine, L407, that is critical for catalysis (Fig. 1) [36]. The rather distal location of Y649C relative to the active site raises the question as to how this mutation might influence h12-LOX activity through structural and/or dynamic regulation. As there is no X-ray structure of the h12-LOX, we pursued hydrogen-deuterium exchange mass spectrometry (HDX-MS). We had previously used HDX-MS to characterize the difference in exchange properties of a h12-LOX mutant, L183E/L187E, which forms an engineered monomeric form of the enzyme [19]. Room temperature HDX-MS helped to identify distinctive exchange properties between the WT dimer and the mutant monomer, thereby providing a map of the dimer interface, which was found to be localized along helix α2 and to serve as the gateway to substrate portal. HDX-MS has been used to study the impact of disease-linked mutations on protein flexibility and dynamics for several other protein systems, and therefore offers an incisive method to examine the structural basis for this SNP of h12-LOX (Y649C) [41-44].

In the current study, using the same set of 45 non-overlapping peptides as previously identified [19], we compared the HDX-MS properties of WT h12-LOX to the current mutant, Y649C h12-LOX, at two temperatures: 10 and 25 °C (Table S1). As described above, there was a notable shift in catalytic activity at 42 °C for WT compared to Y649C (Fig. 2A). However, the functional stability for the WT variant at and above 37 °C (and higher) begins to drop after several minutes (Supporting Information, Fig. S4), precluding reliable HDX analysis [45] of longer incubation times at temperatures ≥37 °C. Thus, we limited our HDX measurements to 10 and 25 °C. The conditions of the described HDX-MS experiments, where exchange proceeds via the EX-2 mechanism [31,46], as indicated by the progressive shift in the isotopic mass spectral envelope, permit a temperature-dependent analysis of protein thermal fluctuations. This property is particularly informative for activity-altering protein mutations, as temperature-dependent HDX-MS can reveal mutation-induced changes on the temperature dependence of regional transient fluctuations of the protein linked to enzyme function [47-49].

Note that non-conserved mutations, as in the case of Tyr-to-Cys, can influence the liquid chromatography (LC) retention times and/or intrinsic back-exchange values for the peptide(s) that contain the site of mutation, which could influence the true exchange properties of a given peptide. For example, the peptide containing the mutation, 645–650, exhibited no significant shift in LC retention time, with ~6.8 and ~6.6 min for the WT h12-LOX and mutant, respectively. However, there was a modest change in the back-exchange value seen for the peptide containing the Cys mutation, with a ~31% value compared to 20% for WT h12-LOX, despite nearly identical LC retention times. Peptide specific back-exchange values, collected for both WT and Y649C, were used to correct for the HDX at each peptide (Supporting Information, HDX Summary Table).

**Fig. 4.**
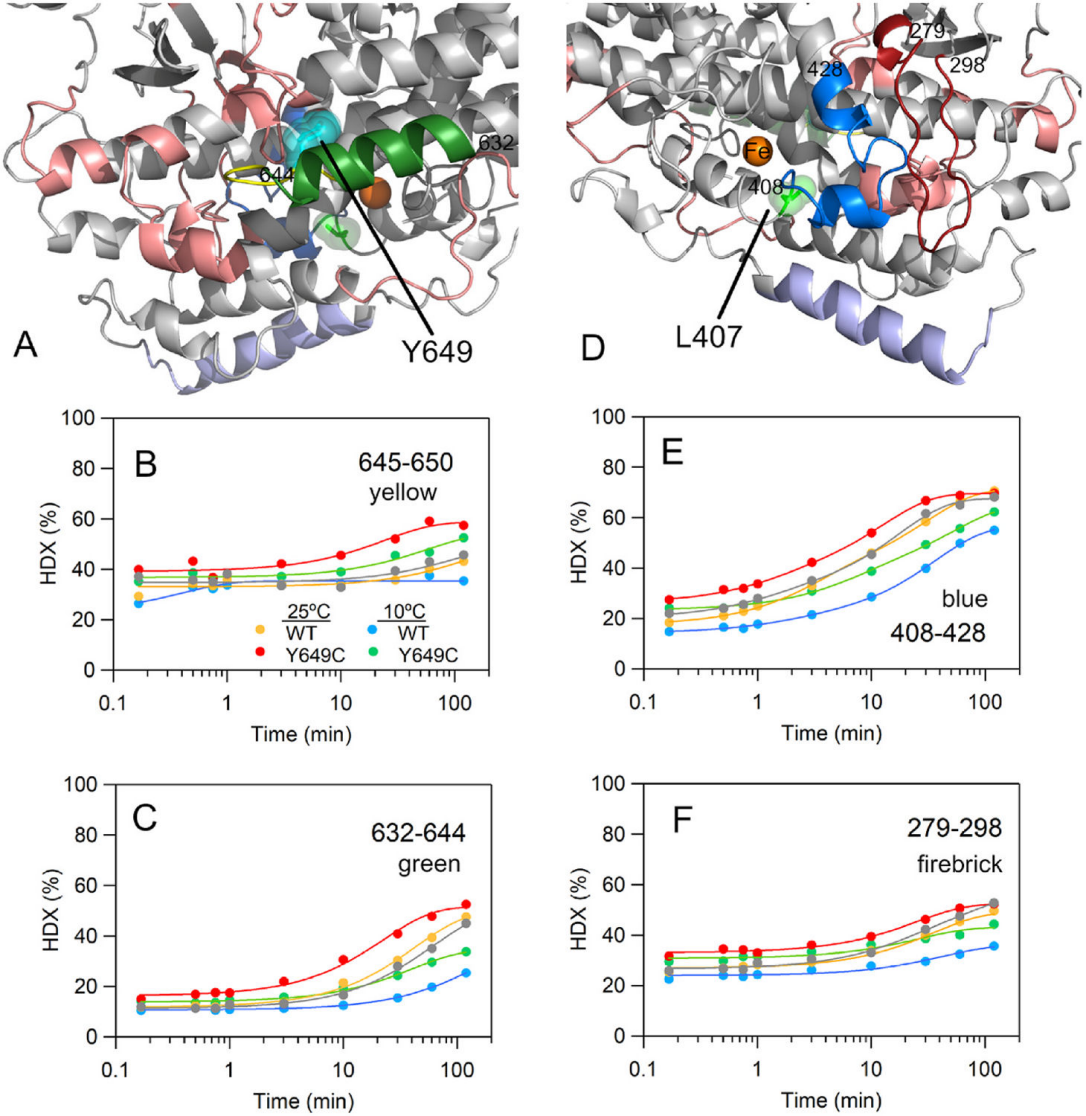
HDX-MS maps (A, D) and representative traces (B, C, E, and F) displaying differences in exchange properties between WT h12-LOX and the Y649C SNP variant of h12-LOX. Y649, the site of mutation, is shown as cyan spheres in (A) for reference. The grey HDX traces in B, C, E, and F represent a biological replicate of WT h12-LOX at 25 °C, which was reproduced from our previous report [19]. The active site residue, L407, is represented by green spheres in (D). In A and D, the numbering of the colored peptides corresponds to the peptides displaying altered exchange properties and are shown in the other panels. The raw MS data for each of these peptides is presented in Figs. S5-S8. The peptides colored in salmon also exhibited altered exchange patterns, as shown in Fig. S9. Helix α2 is colored as light blue and represents the dimer-dimer interface of h12-LOX. In (B–C, E-F), the grey trace represents WT h12-LOX collected at 25 °C from our previous work [19] and represents a biological replicate.

From the inspection of the time dependent HDX traces, there were 12 non-overlapping peptides that exhibited distinct hydrogen exchange behavior for Y649C relative to WT h12-LOX. To be considered a significant difference between variants, at least three time points must deviate by >5% exchange at both temperatures. The most notable difference in exchange properties was centered at the site of mutation, peptide 645–650, particularly at longer incubation times of ≥10 min (Fig. 4A and B). In fact, the extent of exchange for this peptide at nearly every time point for Y649C at 10 °C was elevated relative to the WT values at 25 °C. In addition, the peptide immediately adjacent to the mutation (i.e., 632–644) shows comparable exchange behavior to 645–650 (*cf*. Fig. 4A-C). To further corroborate the impact of the mutation at the mutation site, we also present HDX analysis of two additional peptides that overlap the 645–650 peptide: 632–650 and 635–650 (Supporting Information, Fig. S4). Note that the mutant effect on exchange properties is more prominent for the shorter of the two peptides, 635–650, in which the HDX time-dependent pattern of the Y649C protein at 10 °C nearly matches the exchange property of WT h12-LOX at 25 °C. Because HDX apparent rates and/or extent are often expected to increase with temperature, these data support that the Y649C mutant loosens up the local structure and dynamics of the peptides containing or surrounding the site of mutation.

Further, this behavior is seen to radiate from the site of mutation to several spatially adjacent peptides, ultimately influencing the exchange properties of select peptides on the front face of the enzyme (Fig. 4A and D, salmon). One such peptide is 408–428 (Fig. 4E, blue) which encompasses part of the arched helix 11. An overlapping peptide, 415–428, shows similar behavior (Supporting Information, Fig. S4), corroborating the long-range mutational effect. Altered dynamics at this peptide have potential functional importance as helix 11 contains a fully conserved leucine, L407, in h12-LOX (Fig. 4D, green spheres), that is responsible for positioning the reactive carbon of substrate AA close to the Fe(OH) cofactor for efficient catalysis. The role of L407 in catalysis is underscored by a previous kinetic study that reported *k*_cat_ values decreased by 100-fold for the mutation of this leucine to volume-reducing alanine or glycine [19]. A spatially adjacent peptide, 279–298 (Fig. 4F), also tracks the mutant-induced exchange pattern at 408–428. The former peptide shares hydrophobic packing and some cross-stranded H-bonding with the active site peptide.

It is notable that peptide 186–194, which comprises part of helix α2, also exhibits some slight changes in exchange upon Tyr-to-Cys substitution at 649 (Supporting Information, Fig. S9). However, these effects are modest compared to the dramatic increases in exchange properties previously observed from the monomeric mutant (i.e., L183E/L187E) [19]. Our HDX-MS analysis of Y649C h12-LOX further supports SEC results that the variant maintains the dimeric form and eliminates the possibility of a change in oligomeric state as the origins for the altered kinetic and/or membrane binding behaviors.

### 3.8. Protein stability determined by differential scanning calorimetry

**Fig. 5.**
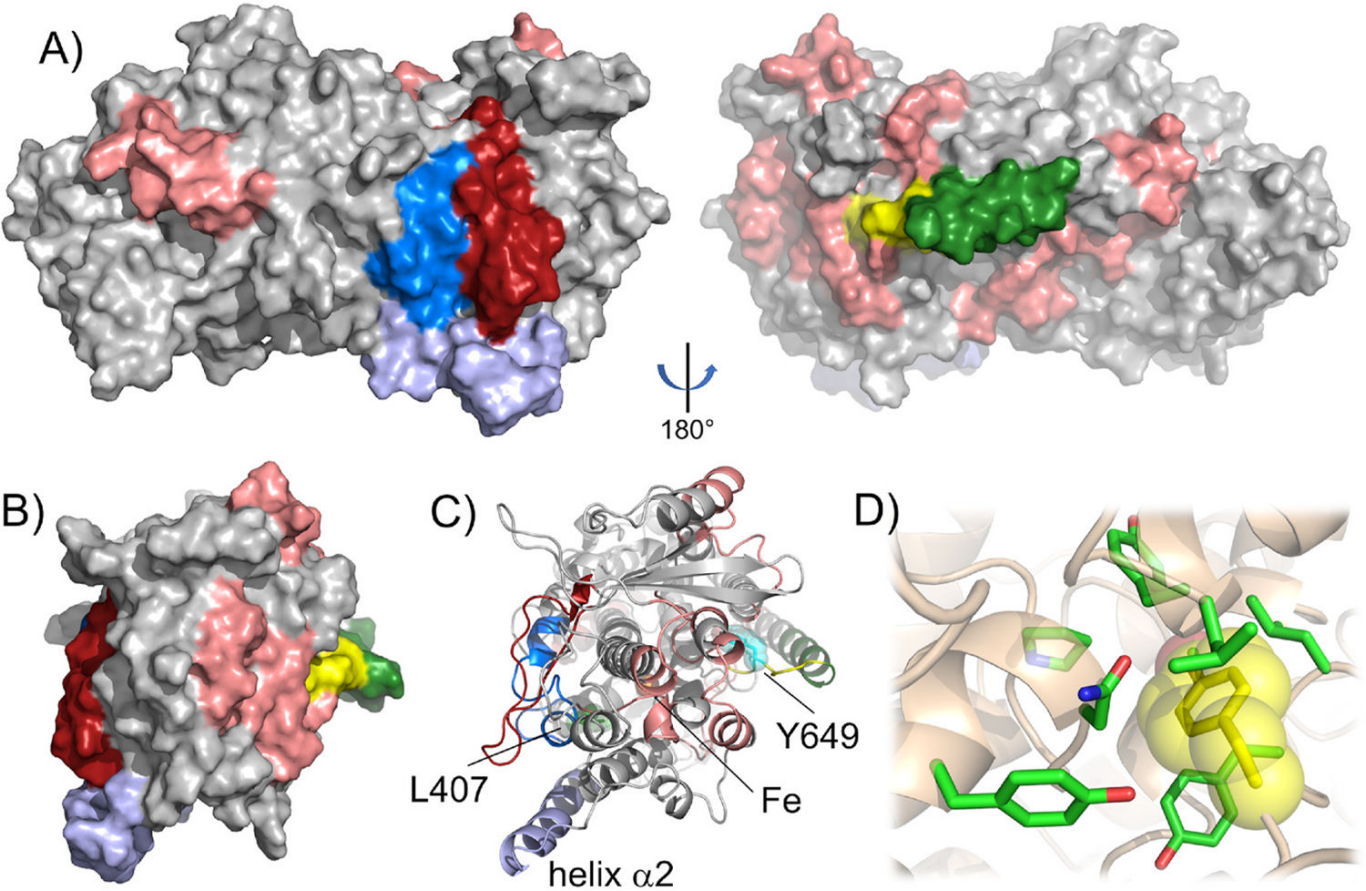
Map of network of h12-LOX derived peptides with altered exchange patterns emerging from the Y649C SNP (A). Panel (B) is rotated 90° with respect to A along the y-axis. Panel (C) represents the cartoon model of space-filling mode from (B) and has the site of mutation, iron center, and L407 represented as spheres and labeled for reference. The peptide coloring is reproduced from Fig. 4. Panel (D) displays the predicted packing environment surrounding Y649, which is represented as yellow spheres.

DSC thermograms of WT h12-LOX produced two distinct T_m_ values of 55 and 58–59 °C, respectively. We assign these thermal transitions to the melting of the dimeric and monomeric species, respectively. Note that these values are slightly elevated relative to the Tm values determined from CD melting traces (WT = 48 ± 0.5 °C and Y649C = 48 ± 1 °C). DSC has the advantage over CD melting traces as the technique is not limited by solution conditions that may influence protein stability. Further, slight differences in the Tm between the two methods may indicate a deviation from a simple two-state unfolding model. Importantly, the WT and Y649C h12-LOX variants produced nearly identical T_m_ values, consistent with the trends in the CD experiment. In addition to the T_m_, DSC also reports on the thermodynamics of thermal unfolding of proteins and is thus complementary to the structural changes that are measured from temperature-dependent CD [50]. DSC has been used to provide quantitative estimates of the structural impact resulting from changes to the sidechain volume on protein (un)folding enthalpy (ΔH°) relative to the native enzyme [51-53]. The Y649C variant shows a significantly reduced ΔH° relative to WT h12-LOX (Table 5). The destabilization of protein inferred from the folding thermodynamics is consistent with the regionally defined enhanced peptide flexibility as detected by HDX, not only at the site of mutation, but extending throughout regions of the protein, including peptides lining the active site (Fig. 5).

### 3.9. Structural and dynamical consequences of Y649C in h12-LOX

For the current study of this h12-LOX SNP, the mutation of Tyr to Cys is a non-conservative, hydrophobic substitution. The mutation does not lead to increased protein aggregation as SEC data support a predominant elution peak corresponding to a dimeric form (Supporting Information, Fig. S3). Protein migration in SDS-PAGE under non-reducing conditions is identical to migration of WT and Y649C under reducing conditions (Supporting Information, Fig. S10), consistent with the expected absence of inter-subunit disulfides resulting from the mutation. The environment surrounding of Y649 (Fig. 5D) is predicted to be primarily hydrophobic. The mutation likely disrupts the packing of these hydrophobic residues through loss of sidechain bulk and/or through infiltration of water molecules, as previously seen for a Tyr-to-Ser mutation in ketosteroid isomerase [54]. In either case, the Y649C mutation in h12-LOX loosens up the peptide to which Y649 resides, thereby eliciting enhanced flexibility of adjacent (and remote) peptides.

Relevant to the current work, a previous mutational study examined the structural and functional consequences of the substitution of a conserved tyrosine, located in the P+1 loop of a Ser/Thr kinase, for a more hydrophobic residue, alanine (i.e., Y204A) [55]. This tyrosine is expected to stabilize the substrate through H-bonding interactions. Loss of this hydrogen bond from the Y204 sidechain, upon Ala mutation, was accompanied by an impaired catalytic rate and altered (enhanced) global dynamic properties of the protein as assessed by NMR and HDX-MS [55,56]. Conversely, there were no notable changes in protein structure through the comparison of X-ray structures [57]. This is a classic example of a dynamic allostery mechanism [58], one in which allostery is mediated without large changes in conformation or oligomerization [59,60]. Notable, the P loop is a hot spot for kinase SNPs linked to disease, in which mutations presumably alter the dynamic landscape of kinases [61].

Herein, the patterns of enhanced exchange stemming from the Y649C mutation of h12-LOX are reminiscent to this dynamic allosteric mechanism [62-64]. Similarly, dynamic allostery has also been inferred for another human LOX isoform for its interaction with a natural anti-inflammatory [65] and for the regulation of SLO-1 by allosteric modulator, oleyl sulfate [66]. The comparative HDX-MS traces for WT and Y649C as a function of time and temperature reveal networks on two faces of the protein surface, one surrounding the site of mutation and another set of peptides proximal to the active site. Note that the connectivity between these two sets of peptides is not immediately apparent (Fig. 5A-C). Typically, HDX-MS reveals the end points of allosteric pathways in proteins since HDX reports on the changes in H-bonding in peptides, with the most dramatic effects arising from regions that are more solvent accessible (i.e., ends of the pathway) [67]. Taken together, the HDX-MS study presented herein for the h12-LOX SNP resulting in a Tyr-to-Cys mutation at position 649 presents a long-range network of peptides with enhanced protein flexibility, extending from the site of mutation to peptides as far as 40–50 Å away and includes those lining the active site (Fig. 5). Our results expand upon previously characterized dynamic ‘allosteric-like’ networks of catalysis-altering peptide flexibility originating from protein mutations that have been detected by mutant-dependent HDX-MS for several other systems, such as TIM barrels [48,68], kinases [56,69], thrombin [67, 70], and tRNA synthetases [71].

## 4. Conclusions

An SNP of h12-LOX, Y649C, was discovered in a patient suffering from a bleeding disorder stemming from impaired platelet aggregation [26]. It was proposed that this clinical symptom was due to a lowering of the enzymatic rate of the Y649C in the platelet but the molecular basis of the lower function was unclear. To investigate this *in vivo* observation, Y649C was expressed/purified, and its biochemical properties were determined. Y649C manifested a similar preference for AA catalysis over that of DHA and displayed comparable product profiles for both AA and DHA. The inhibitor response to ML355 was also similar to WT, suggesting similar active site structure with respect to inhibitor constraints. Synthetic membrane affinity of Y649C was also comparable to WT displaying a preference (PC and PE over PS) and a modest calcium effect. Y649C did display a greater membrane affinity than WT h12-LOX at 22 °C, however, at 37 °C its affinity was slightly lower than WT. The steady-state kinetic parameters of Y649C were lower than that of WT at 37 °C, with *k*_*cat*_ being over 6-fold lower, however, their second order rate constants were comparable at this temperature, indicating that the substrate concentration in the cell is key to establish which kinetic parameter affects cellular activity more. Interestingly, Y649C displayed greater catalytic stability to temperature, however, its protein stability as measured by DSC was significantly lower than that of WT. Finally, H/D exchange measured greater peptide mobility near the site of mutation that propagated to the active site for Y649C compared to WT, providing a structural basis for the dynamic, allosteric-like effects stemming from this SNP. In summary, many of the biochemical traits of Y649C were similar to WT h12-LOX at 37 °C, except for lowered kinetic parameters, lowered synthetic membrane affinity, and enhanced regional peptide flexibility associated with decreased structural stability. Considering that h12-LOX most likely obtains its fatty acid substrate from the phospholipid bilayer and the biological temperature is 37 °C, it is possible that the lower activity of Y649C observed in patient platelets could be due to a combination of its lower activity, lower membrane affinity and its lower protein folding stability.

## Supplementary Material

supplement

## Figures and Tables

**Table 1:** Steady-State kinetic parameters for WT h12-LOX and Y649C at three temperatures. Standard deviation errors are shown in parentheses from 3 replicates.

	*k*_*cat*_ (sec^−1^)		*k*_*cat*_/*K*_*M*_ (μM^−1^sec^−1^)	
Temperature (°C)	WT	Y649C	WT	Y649C
22	22 (4)	2.6 (6)	10 (0.2)	0.76 (0.4)
37	21 (1)	3.6 (0.1)	8.7 (2)	6 (0.6)
42	16 (0.6)	12 (0.3)	16(3)	2.6 (0.2)

**Table 2:** Competitive kinetic product distribution with a mixture of 10 μM AA and 10 μM DHA with both WT h12-LOXand Y649C. Standard deviation errors are shown in parentheses from 3 replicates.

	AA products (%)	DHA products (%)
WT h12-LOX	65 (6)	35 (3)
Y649C	63 (6)	37 (4)

**Table 3:** Distribution of products created by reaction of either WT h12-LOX or Y649C with AA or DHA (10 μM). Percent values are presented. Standard deviation errors are shown in parentheses from 3 replicates.

	AA Products		DHA Products			
	12- HETE	15- HETE	14- HDHA	17- HDHA	20- HDHA	11- HDHA
WT	88 (2)	12(2)	76 (2)	11 (0.5)	0	13 (2)
Y649C	81 (1)	19(1)	74 (2)	8(2)	8(1)	10(1)

**Table 4:** I_50_ values and maximum inhibition percentage of ML355 against WT h12-LOX and Y649C. Standard deviation errors are shown in parentheses from 3 replicates.

	IC_50_ μM (error)	Maximum % inhibition
WT	0.9 (0.2)	91 (5)
Y649C	1.1 (0.2)	90 (4)

**Table 5:** DSC thermodynamics for WT h12-LOX and Y649C h12-LOX unfolding.

	T_m,dimer_ (°C)	T_m,monomer_ (°C)	ΔH° (kJ/mol)
WT	55	58–59	1200
Y649C	55	60–61	350

## Abbreviations

LOX: lipoxygenase
h12-LOX: human platelet 12S-lipoxygenase
r15-LOX-1 or r12/15-LOX or rALOX15: rabbit 15S-LOX-1
c11-LOX: coral 11R-LOX
AA: arachidonic acid
DHA: docosahexaenoic acid
12(S)-HpETE: 12(S)-hydroperoxyeicosatetraenoic acid
12(S)-HETE: 12(S)-hydroxyeicosatetraenoic acid
COX: cyclooxygenase
ML355: h12-LOX specific inhibitor
NSAIDs: nonsteroidal anti-inflammatory drugs
Coxibs: COX-2 selective inhibitors
13- HpODE: 13S-hydroperoxyoctadeca-9Z, 11E-dienoic acid
13-HODE: 13S-hydroxyoctadeca-9Z,11E-dienoic acid
TOP: a subdomain region in the helical bundle of the catalytic domain (residues 163–222)
PLAT domain: Polycystin-1, Lipoxygenase, Alpha-Toxin
PDZ domain: PSD95, Dig1, Zo-1 domain
E_cat_: catalytically active enzyme
E_apo_: inactive Fe-free enzyme
SAXS: small angle X-ray scattering
ICP-MS: inductively coupled plasma mass spectroscopy
SEC: size exclusion chromatography
BSA: bovine serum albumin
DCM: dichloromethane
HDX-MS: hydrogen-deuterium exchange-mass spectrometry
WT h12-LOX: wild-type human platelet 12S-lipoxygenase
h15-LOX-2: human epithelial 15-lipoxygenase
SLO-1: soybean lipoxygense-1
SDS-PAGE: sodium dodecyl sulfate-polyacrylamide gel electrophoresis
DOPC: 1,2-dioleoyl-*sn*-glycero-3-phosphocholine
DOPE: 1,2-dioleoyl-*sn*-glycero-3-phosphoethanolamine
DOPS: 1,2-dioleoyl-*sn*-glycero-3-phospho-l-serine
