# Supplementary material for: Biochemical and hydrogen-deuterium exchange studies of the single nucleotide polymorphism Y649C in human platelet 12-lipoxygenase linked to a bleeding disorder
Source: Arch Biochem Biophys. Author manuscript; Available in PMC 2023 Jan 31. (PMC9888433; doi:10.1016/j.abb.2022.109472)
Supplement: supplement [file NIHMS1864854-supplement-supplement.docx]

**Supporting Information**

**Biochemical and hydrogen deuterium exchange studies of the single nucleotide polymorphism Y649C in human platelet 12-lipoxygenase linked to an impaired bleeding disorder**

Michelle Tran,^1^ Adriana Yamaguchi,^2^ Eefie Chen,^1^ Michael Hollinstat,^2^ Rachel L. Signorelli,^3^ Anthony T. Iavarone^4^, Adam R. Offenbacher,^3^* Theodore Holman^1^*

^1^Department of Chemistry and Biochemistry, University of California Santa Cruz, Santa Cruz, CA 95064, United States

^2^ Department of Pharmacology, University of Michigan Medical School, Ann Arbor, MI, 48109

**^3^**Department of Chemistry, East Carolina University, Greenville, NC 27858, United States

^4^QB3/Chemistry Mass Spectrometry Facility, University of California Berkeley, Berkeley, CA 94720, United States

Funding: NIH:AG047986 (TRH), NSF:2003956 (ARO) and NIH:1S10 OD020062 (QB3/Chemistry Mass Spectrometry Facility at UC Berkeley).

***Corresponding Authors:**

TRH: Tel.: +1-831-459-5884, [holman@ucsc.edu](mailto:holman@ucsc.edu)

ARO: Tel.: +1-252-737-5422, offenbachera17@ecu.edu

**Table S1.** HDX data summary for WT and Y649C 12-LOX

| **Data sets** | WT, Y649C 12-LOX |
| --- | --- |
| **HDX Reaction Details** | Labeling conditions: 5 µM protein, 90% D_2_O, 10 mM HEPES, 150 mM NaCl, 5 mM DTT, pD = 7.4; corrected pD = pH_read_ +0.4.  0s timepoint was collected with 5 µM protein, 10 mM HEPES, 150 mM NaCl, 5 mM DTT, pH = 7.4.  HDX was conducted at two different temperatures: 10 and 25˚C |
| **HDX Time Course** | 10 time points (0, 10, 30, 45, 60, 180, 600, 1800, 3600, 7200 s) for each temperature |
| **HDX Controls** | Maximally-labeled control (WT and Y649C) |
| **Back Exchange:**  **Average, Interquartile** | 21.3%, 17.4% for WT  22.7%, 15.8% for Y649C |
| **Number of peptides** | 181 total; 45 chosen for analysis |
| **Sequence coverage** | 90% of peptides were measurable |
| **Average Peptide Length** | 13 amides (5-28) |
| **Replicates** | 1 (biological) for the mutant and temperature.  Each time point for each temperature for each protein was collected once for the mutant and WT at 10°C. 2 biological replicates were collected for the WT at 25°C. To mitigate systematic errors, each temperature set (10 time points of one protein) was collected over three days in a non-sequential order. The temperature dependence provides a check for outlying datasets relative to the WT protein. |
| **Repeatability** | There were replicates for time points in the WT 12-LOX sample collected at 25°C, but other time points were only collected once. However, in majority of the peptides, the data points for WT and Y649C are nearly identical, with only 12 of 45 non-overlapping peptides showing a difference of >5% exchange (>0.5Da difference in mass). |
| **Significant Differences in HDX** | To be considered a significant difference between WT and Y649C mutant, there was a threshold of at least 3 time points showing a HDX percentage difference of at least 5% across both temperatures. |


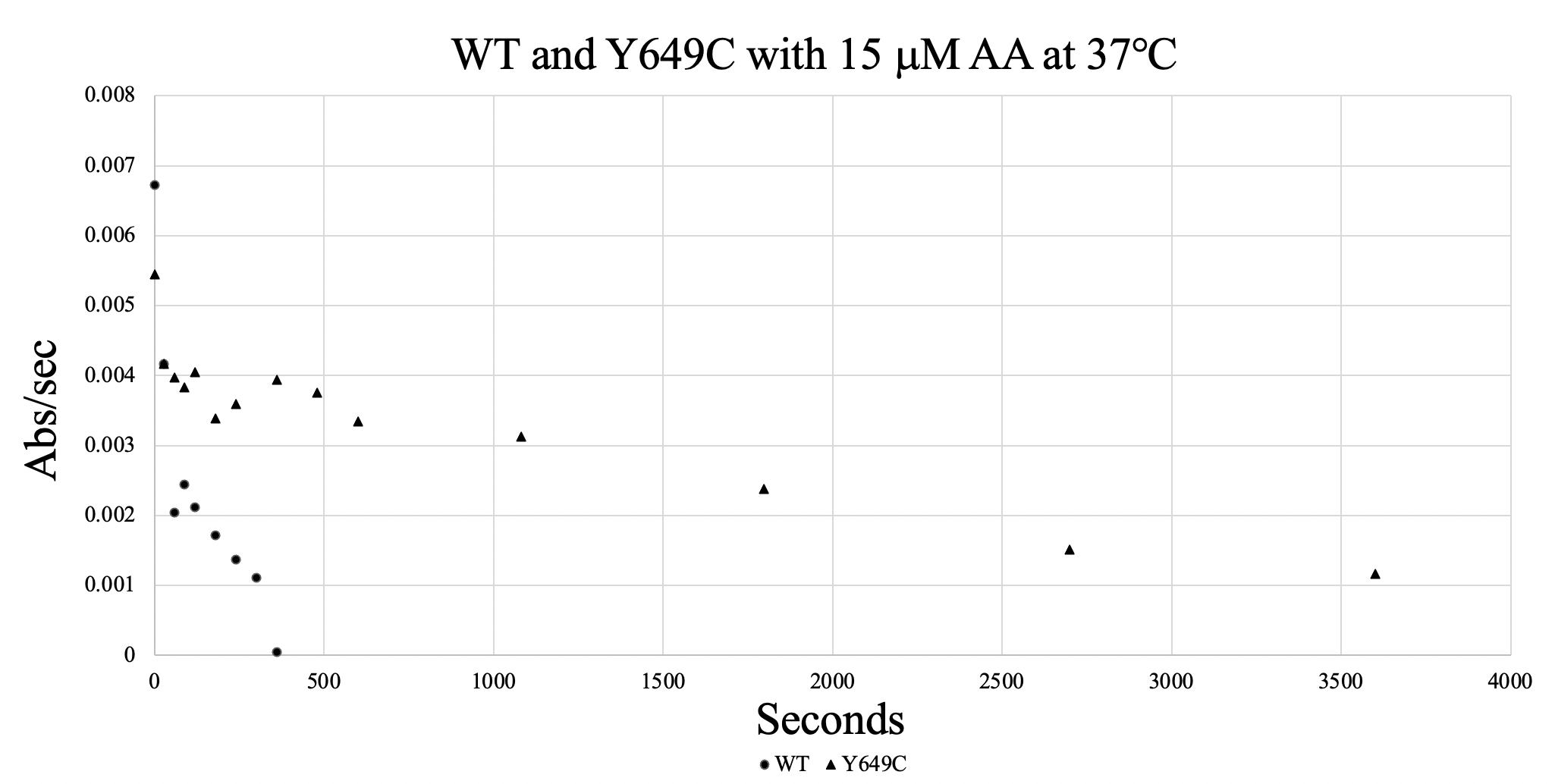


**Figure S1:** Temperature dependence of enzymatic activity for WT and Y649C at 37 degrees C and 15 μM AA.

**
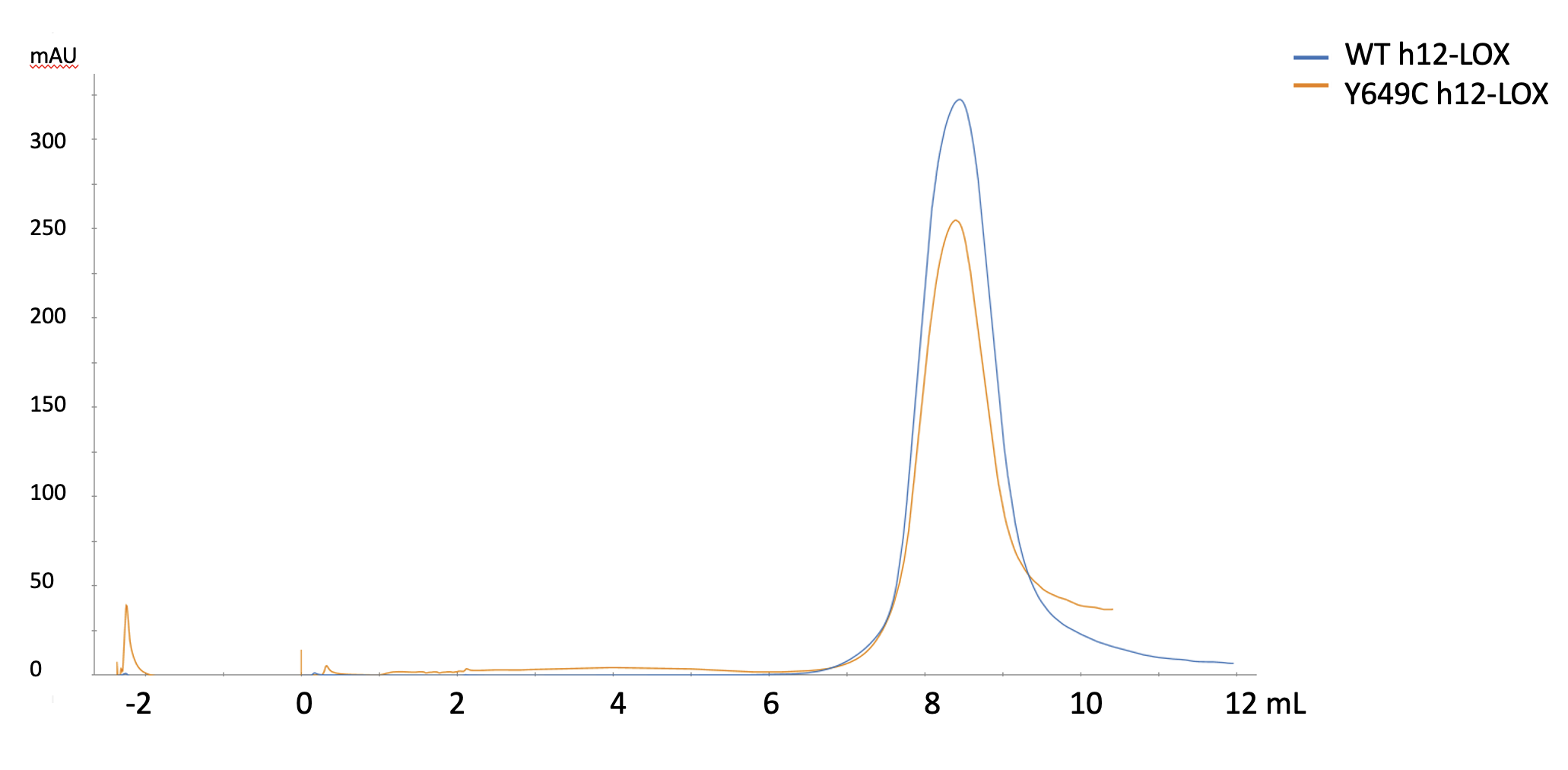
**

**Figure S2:** Size exclusion chromatogram of WT and Y649C demonstrating dimeric form for both enzymes.

A


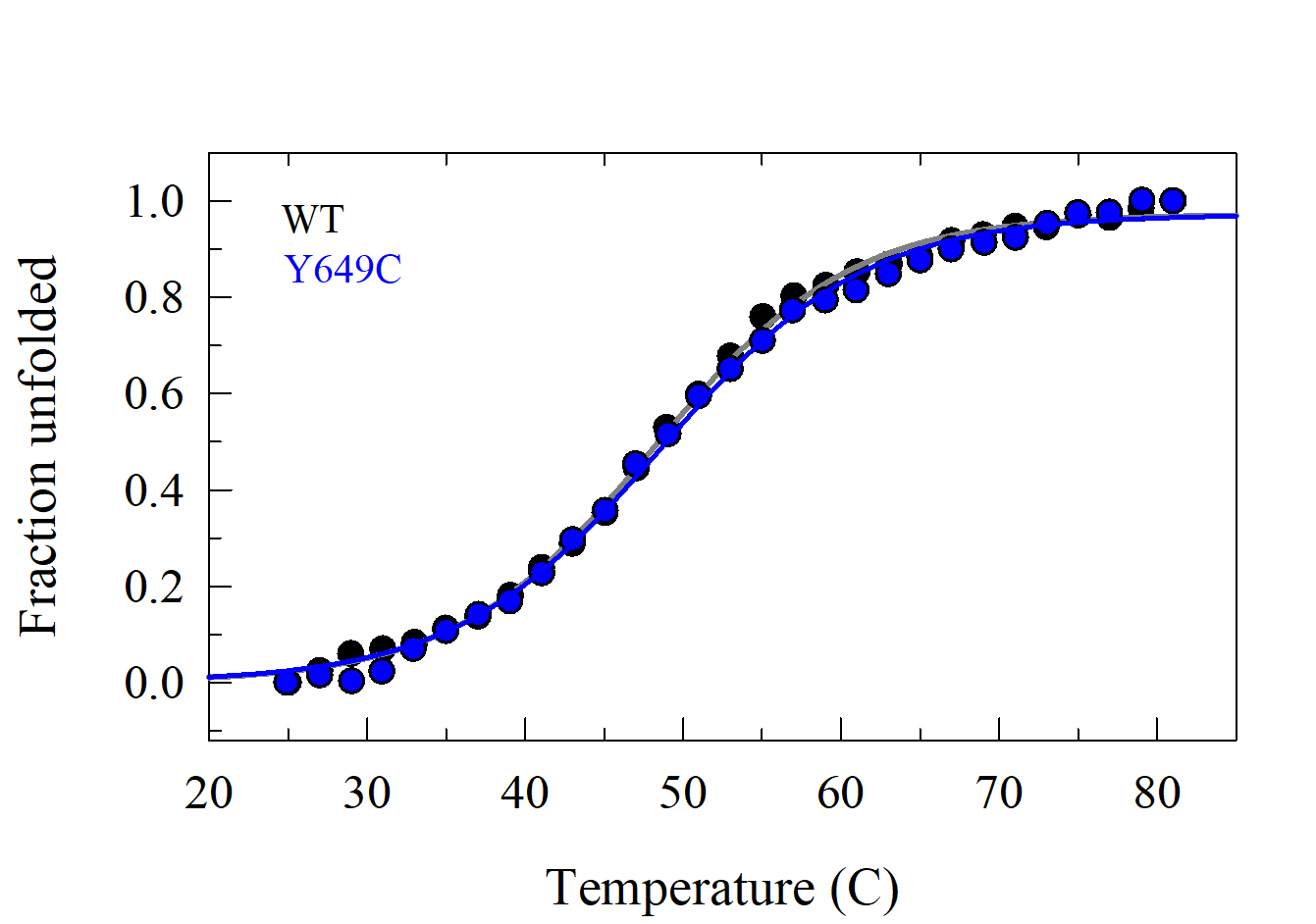


B


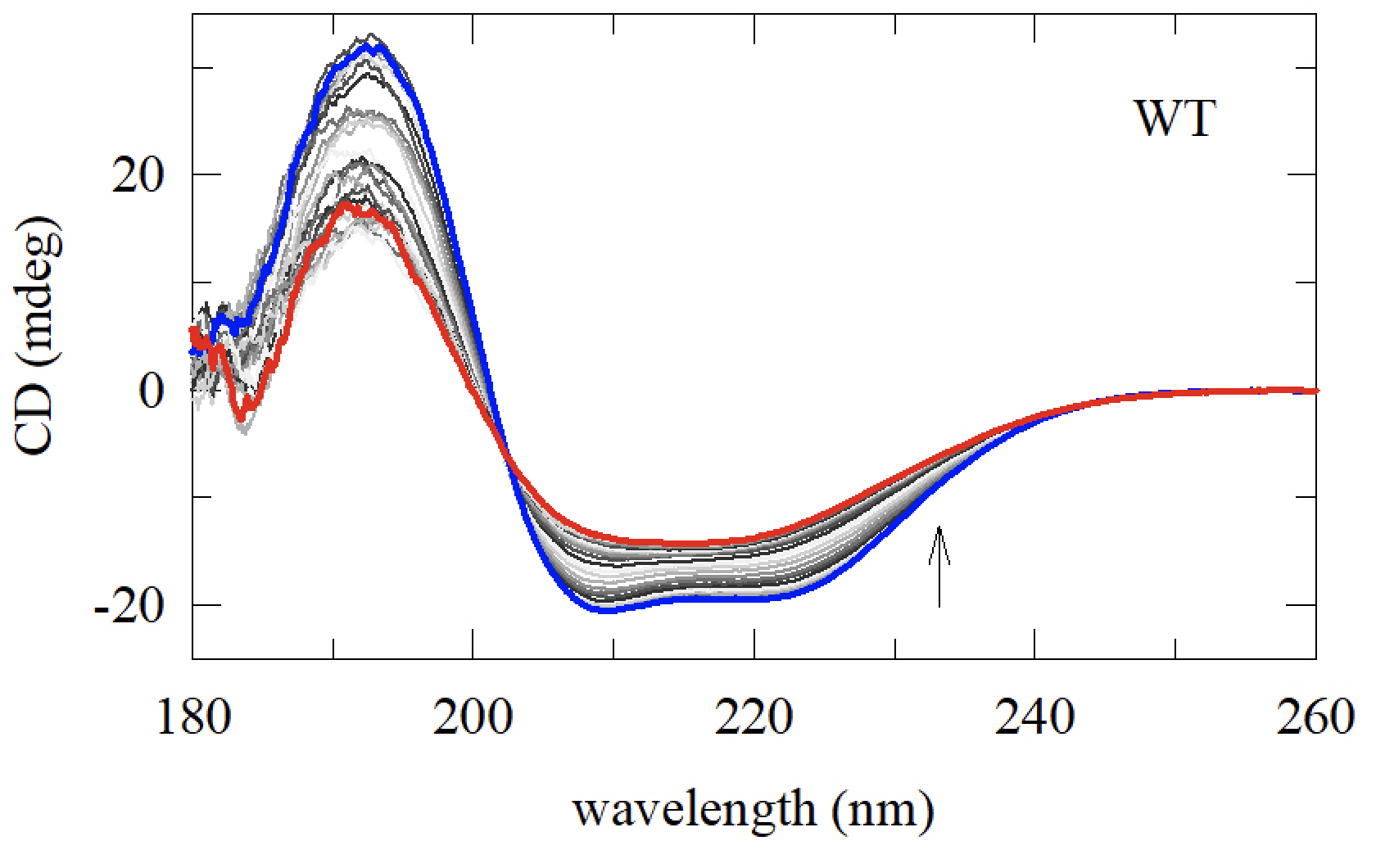


C


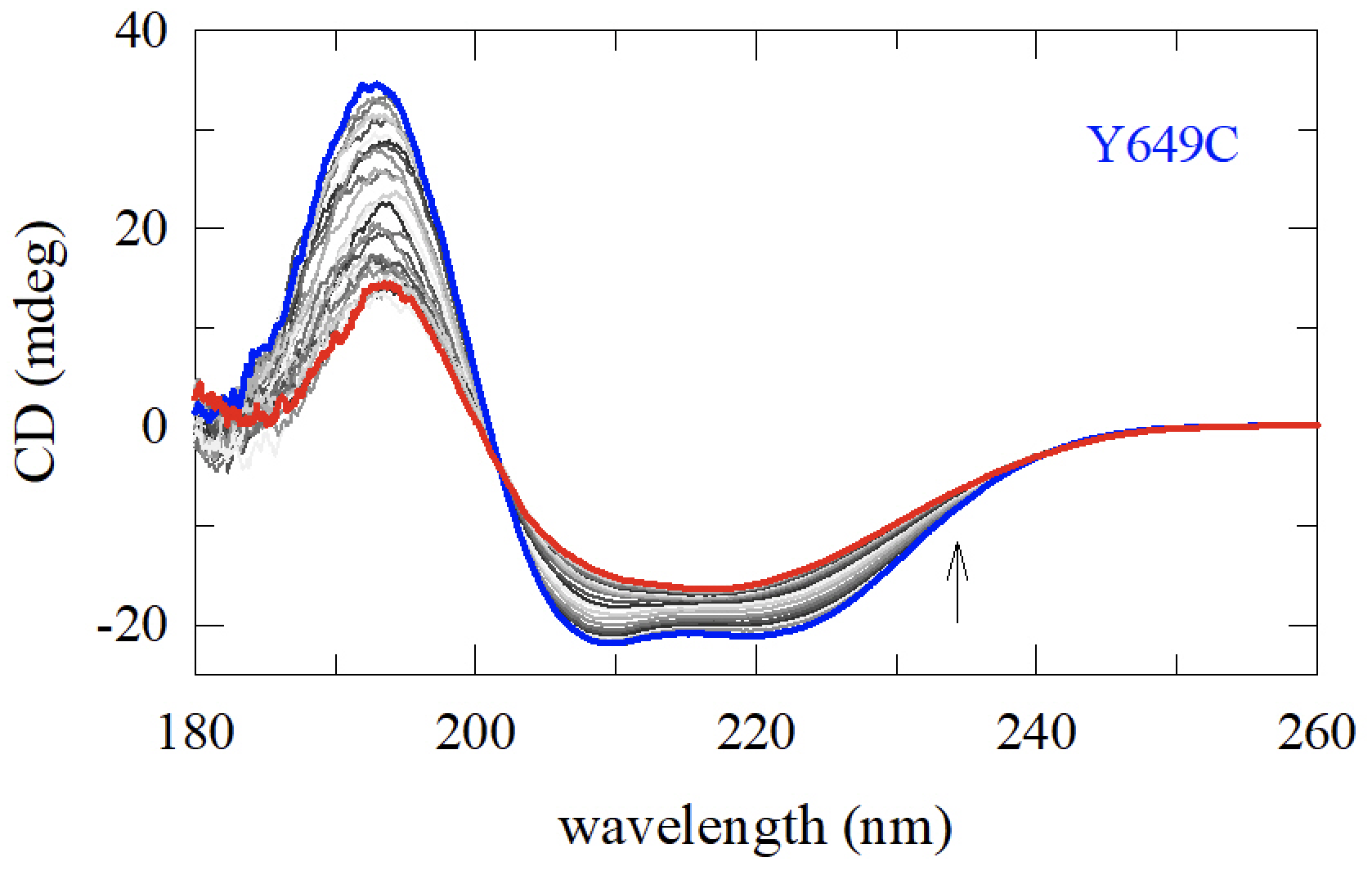


**Figure S3**. **A**. Thermal unfolding of h12-LOX WT (black) and Y649C (blue) as a function of temperature. These unfolding data were obtained by averaging the measured spectral data from 205 to 225 nm (Figure SZ). Fitting the data to sigmoidal functions gives T_m_’s of 48 ± 0.5 ° C and 48 ± 1 °C for the WT and Y649C proteins, respectively. **B**. CD spectra for h12-LOX WT. **C**. CD spectra for h12-LOX Y649C.


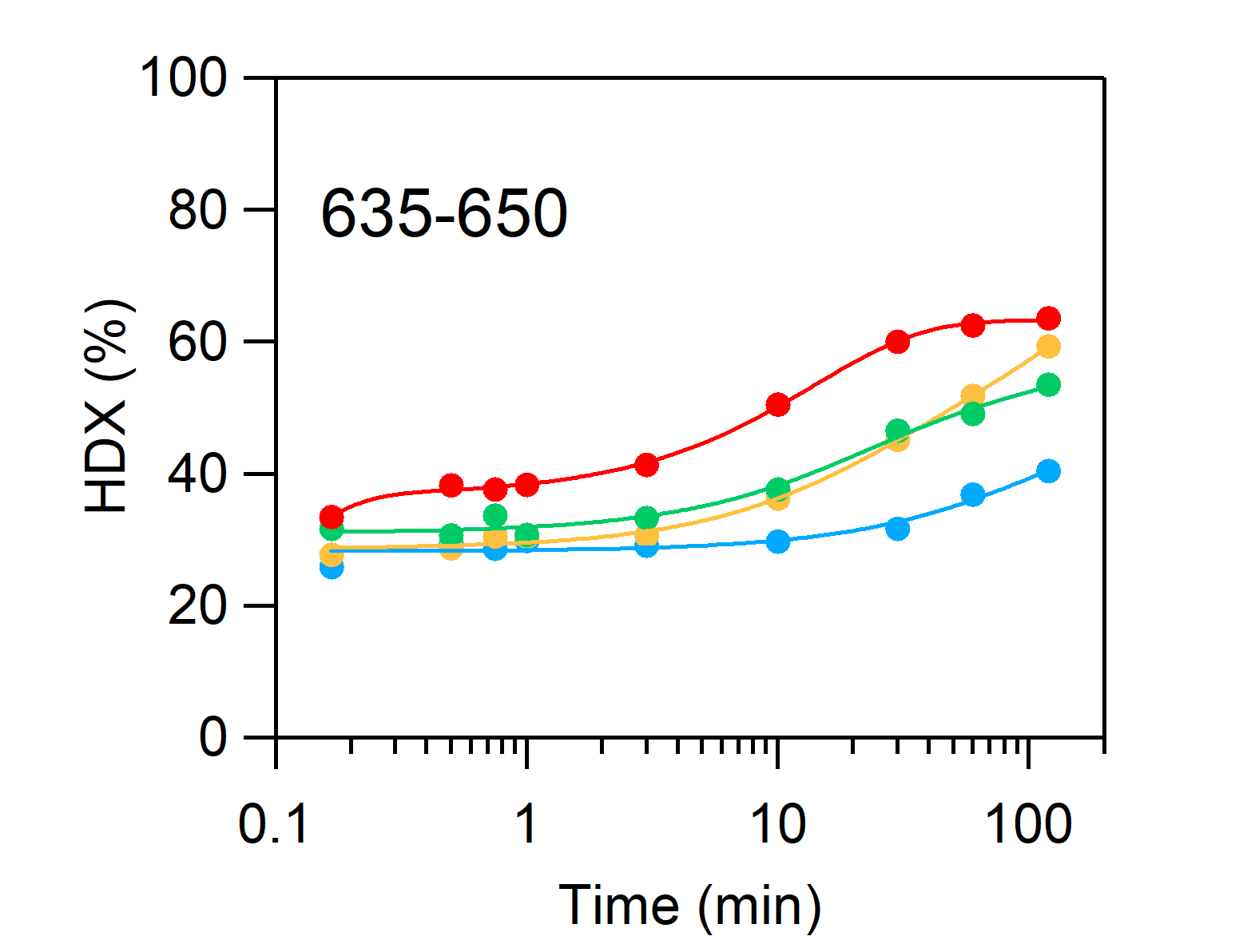


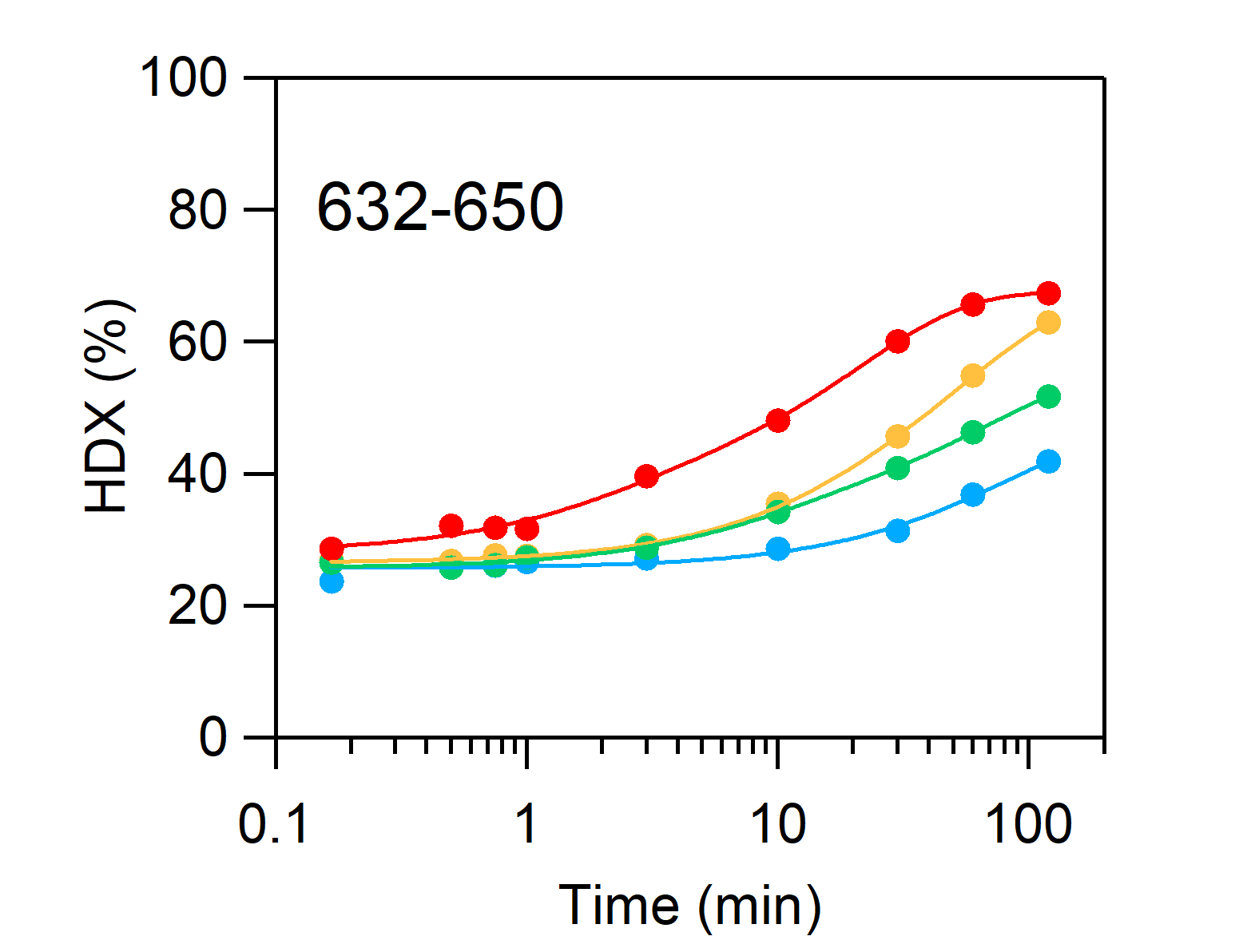


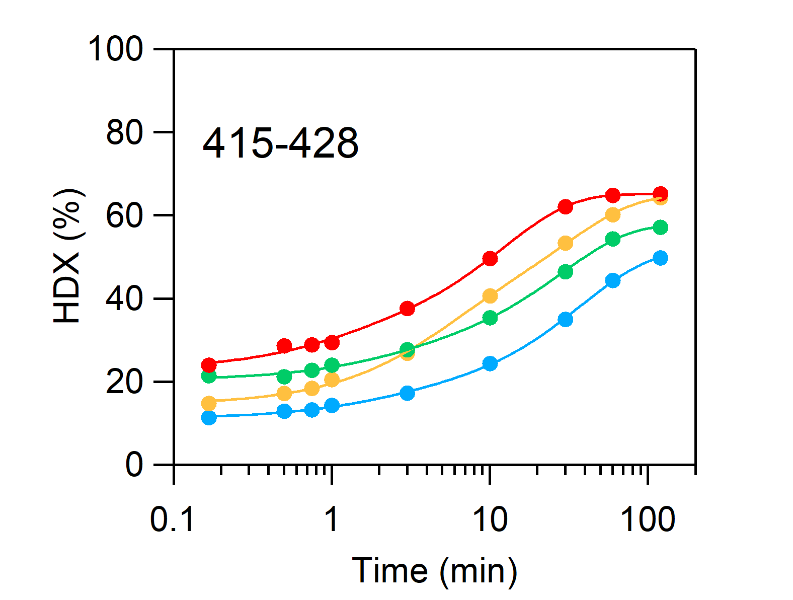


**Figure S4**. Temperature dependence of select overlapping peptides for WT and Y649C h12-LOX. The color coding represents: red, Y649C 25°C; orange, WT 25°C; green, Y649°C; and blue, WT 10°C.

**
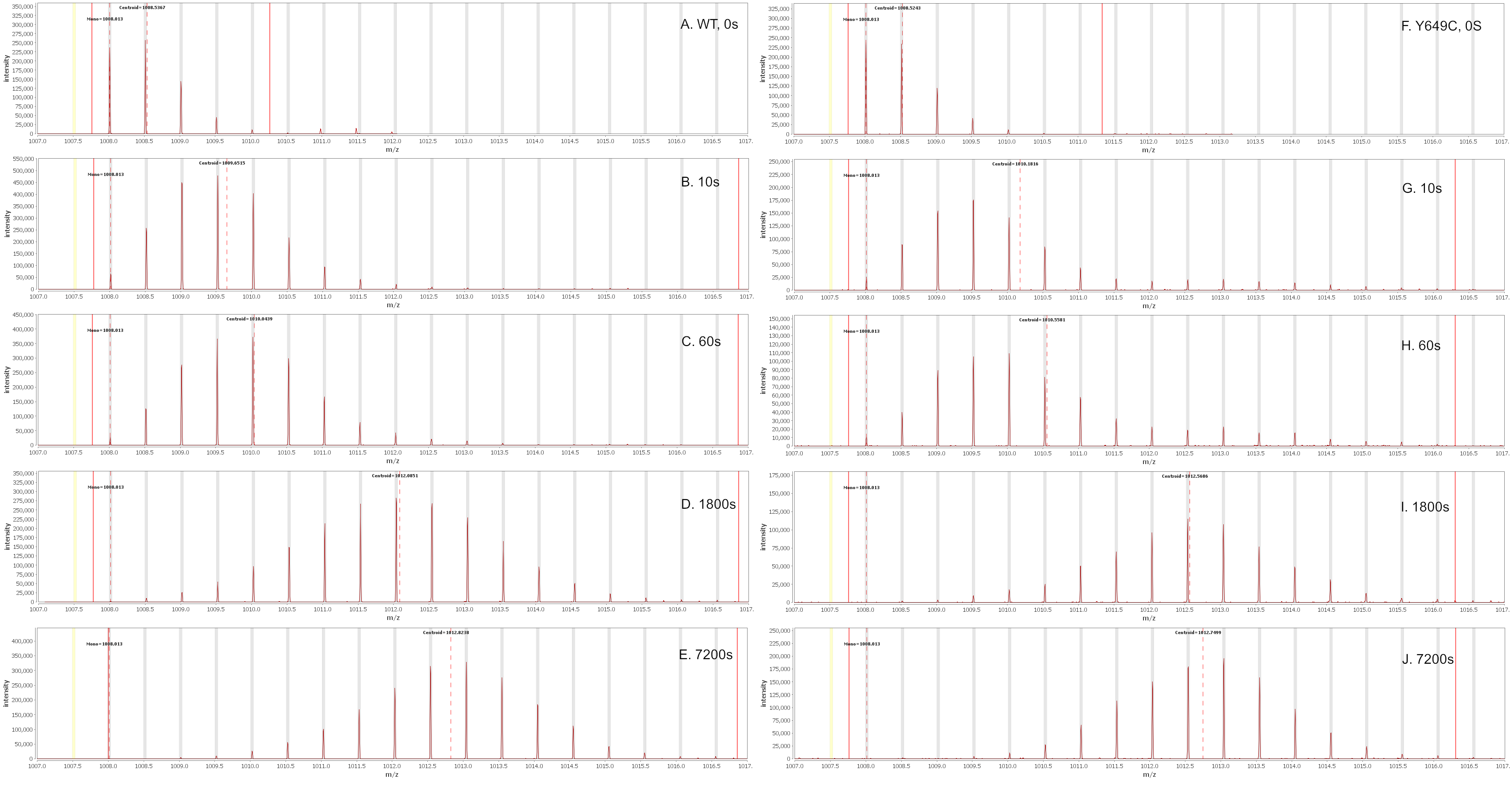
**

**Figure S5**. Mass spectra for peptide 408-428 derived from (A-E) WT or (F-J) Y649C 12-LOX, as a function of time.


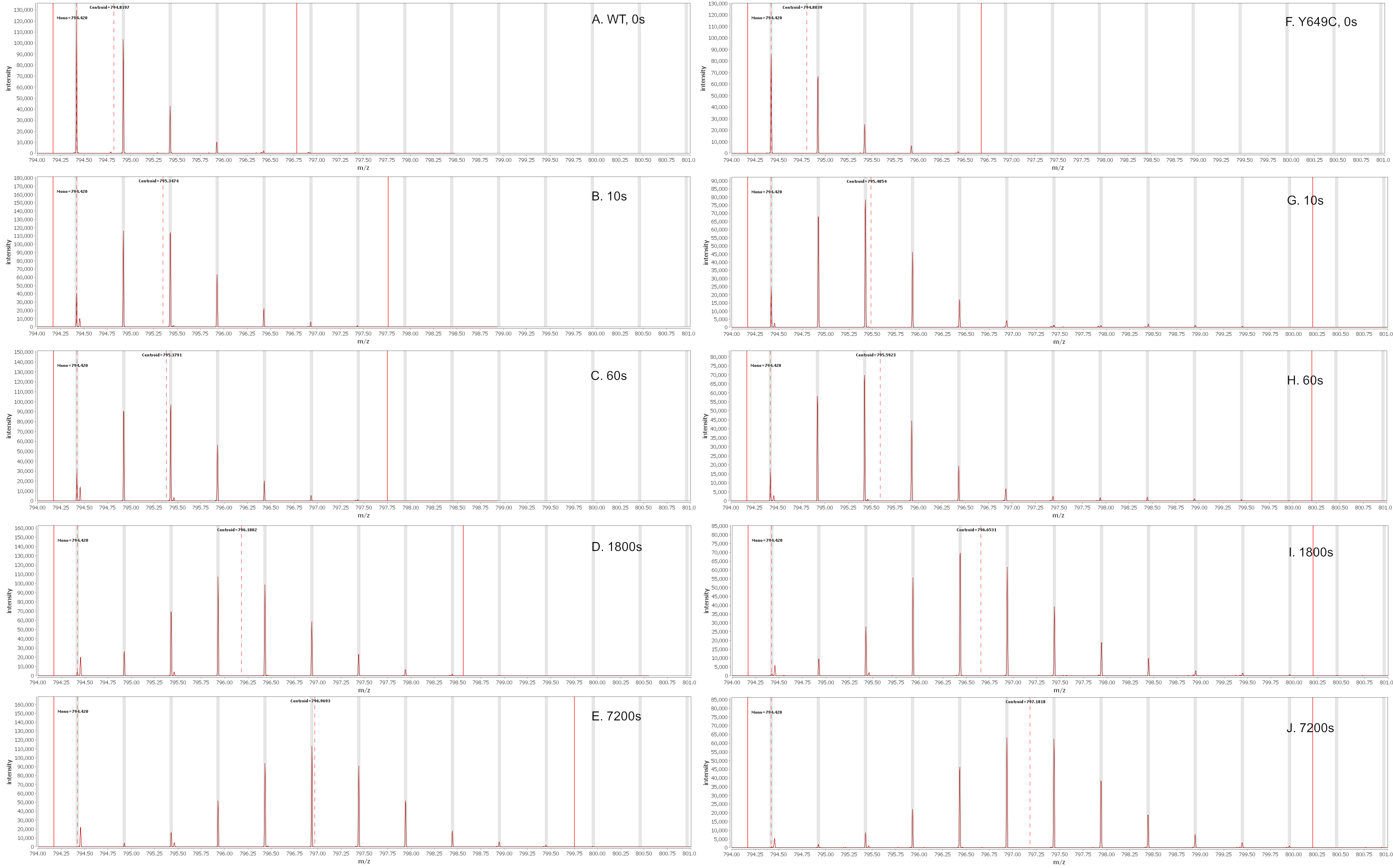


**Figure S6**. Mass spectra for peptide 632-644 derived from (A-E) WT or (F-J) Y649C 12-LOX, as a function of time.


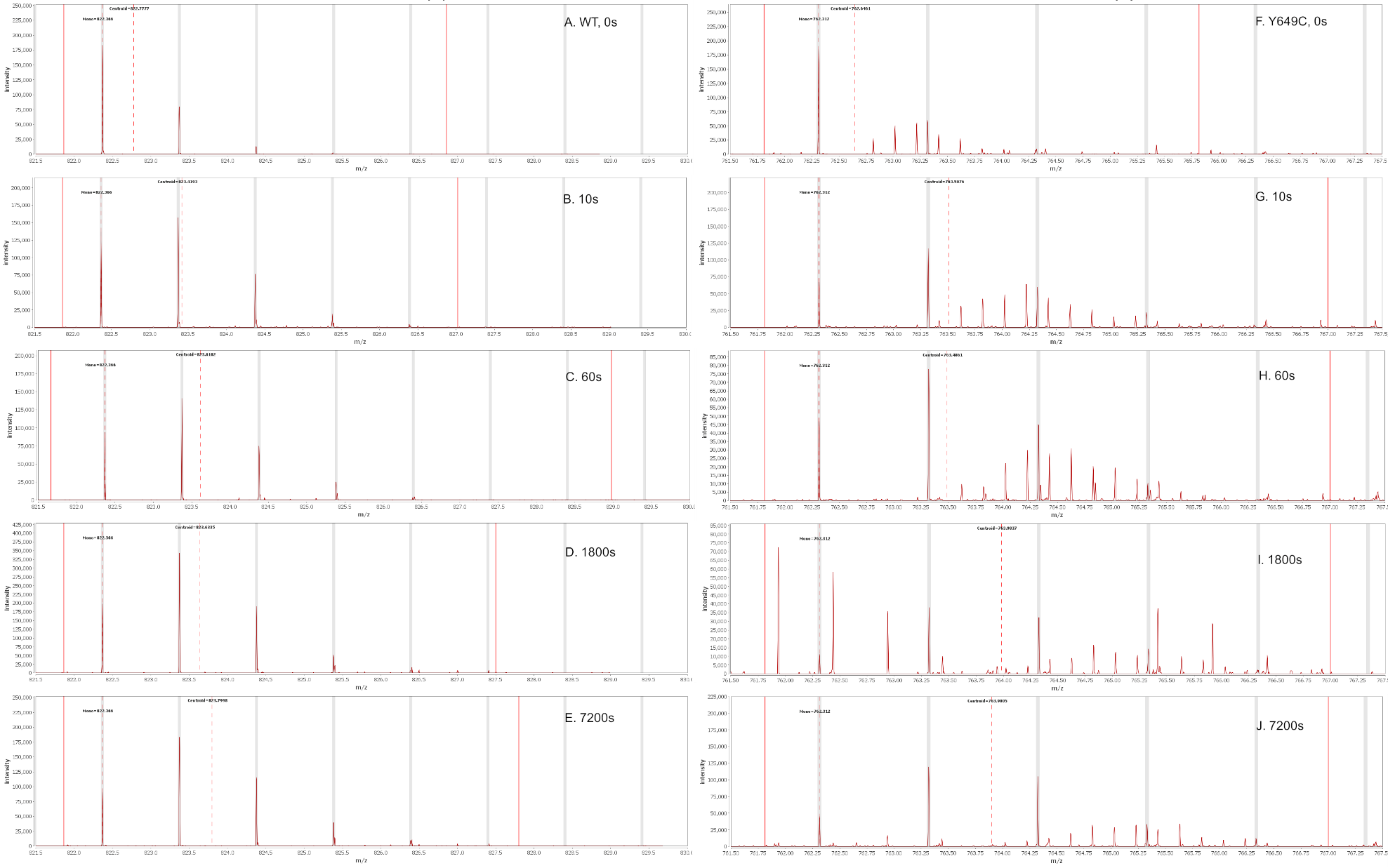


**Figure S7**. Mass spectra for peptide 645-650 derived from (A-E) WT or (F-J) Y649C 12-LOX, as a function of time.


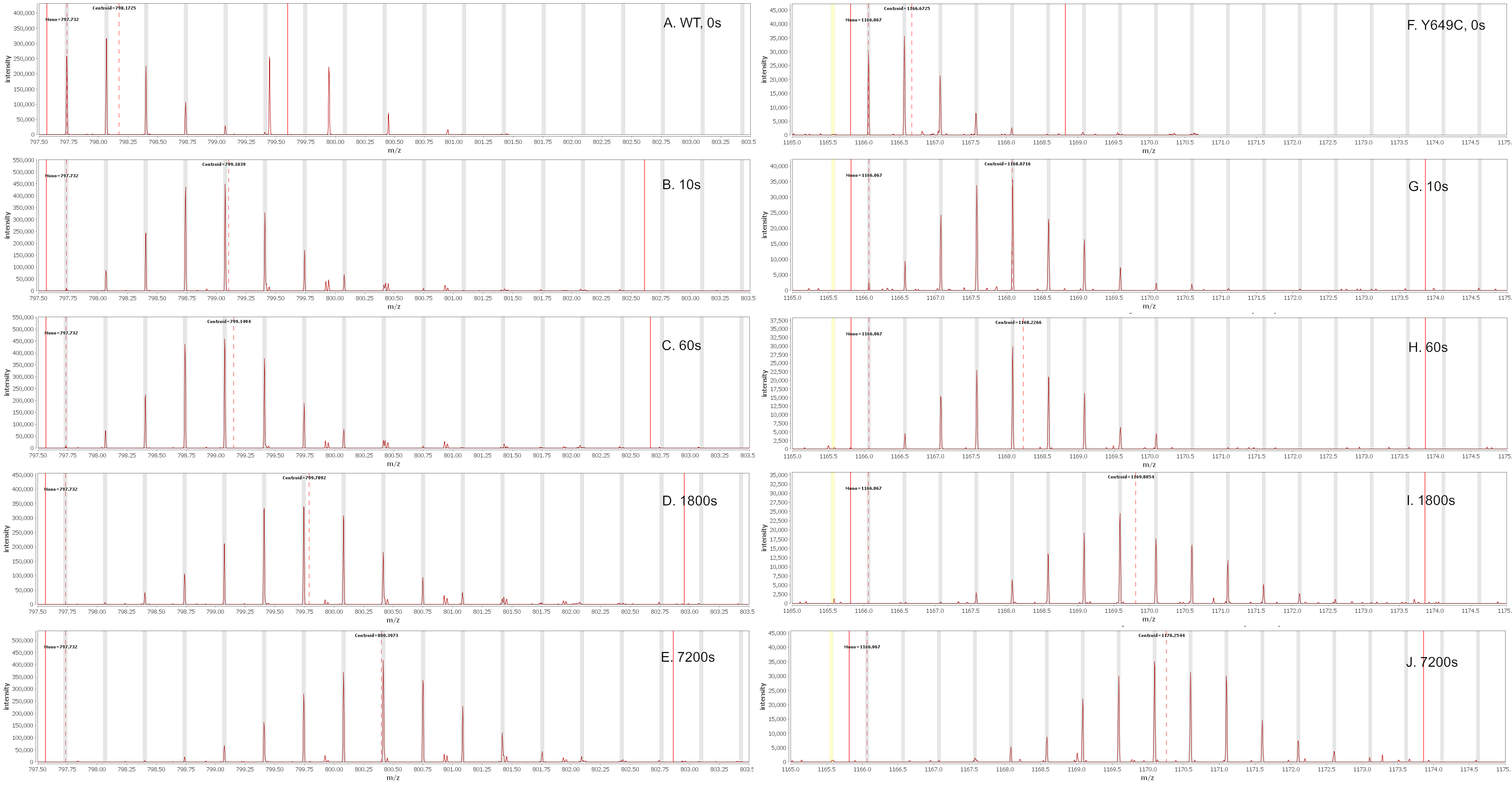


**Figure S8**. Mass spectra for peptide 632-650 derived from (A-E) WT or (F-J) Y649C 12-LOX, as a function of time.

**
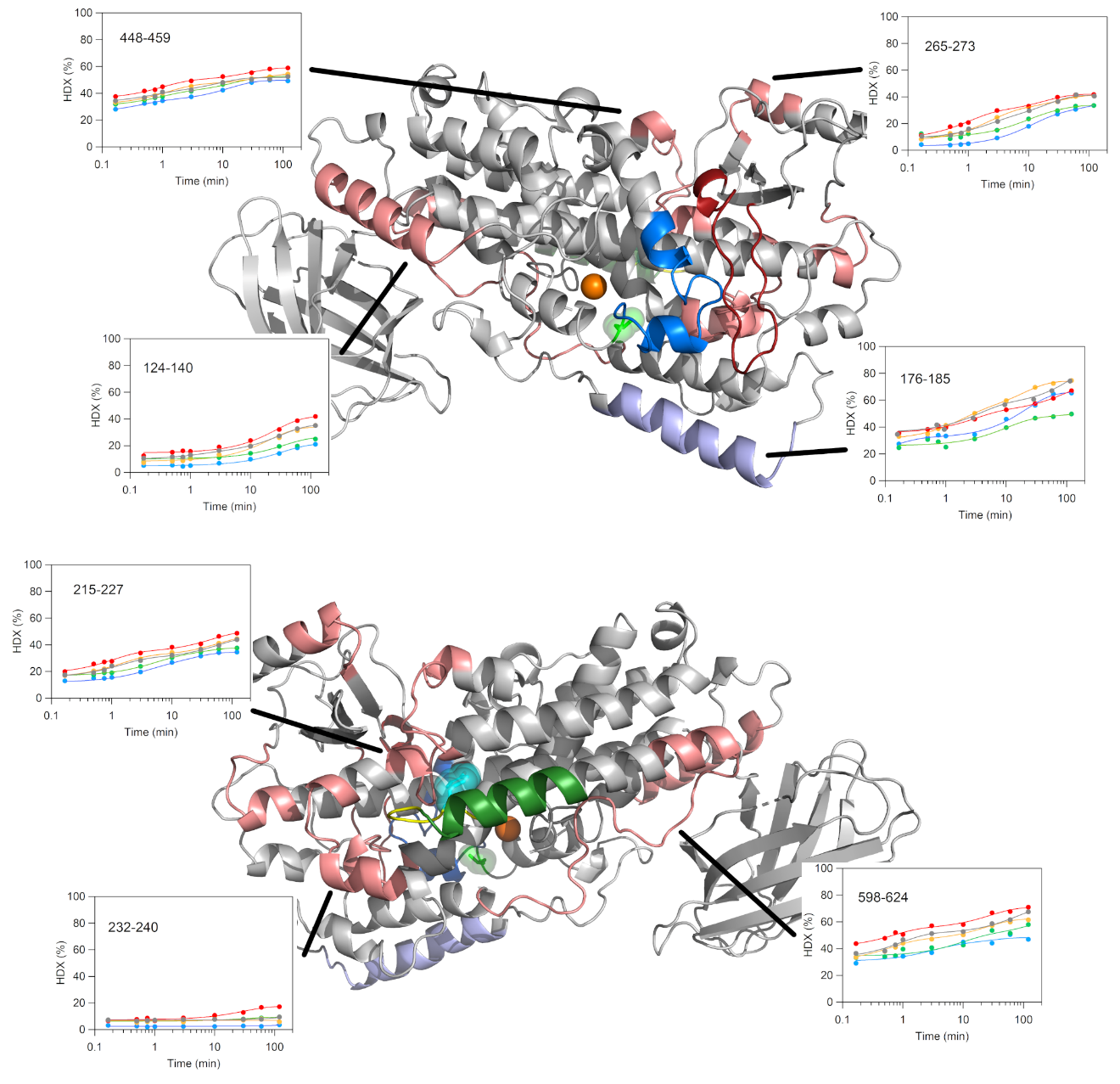
**

**Figure S9.** HDX maps of additional non-overlapping peptides with Y649C-induced altered exchange properties.


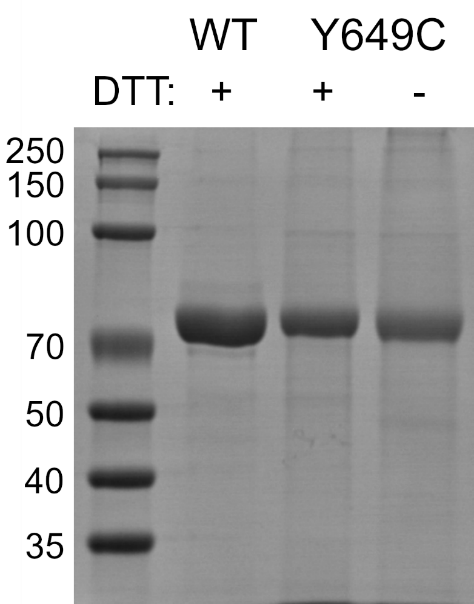


**Figure S10.** SDS-PAGE analysis of WT and Y649C 12-LOX under reducing (+DTT) and non-reducing (-DTT) conditions
